# Dual targeting of BCMA and SLAMF7 with the CARtein system: chimeric antigen receptors with intein-mediated splicing elicit specific T cell activation against multiple myeloma

**DOI:** 10.3389/fimmu.2025.1613222

**Published:** 2025-07-31

**Authors:** Noelia Moares, Pablo Gonzalez-Garcia, Wenjie Yi-He, Juan P. Muñoz-Miranda, Antonio Gabucio, Rosa Luna-Espejo, Javier Ocaña-Cuesta, Ricardo Fernandez-Cisnal, Cecilia M. Fernandez-Ponce, Francisco Garcia-Cozar

**Affiliations:** ^1^ Department of Biomedicine, Biotechnology and Public Health, Faculty of Medicine, University of Cadiz, Cadiz, Spain; ^2^ Institute of Biomedical and Innovation Research Cadiz (INIBICA), Cadiz, Spain

**Keywords:** chimeric antigen receptor, multiple myeloma, inteins, protein splicing, B-cell maturation antigen, SLAMF7, modular CAR, immunotherapy

## Abstract

**Introduction:**

Chimeric antigen receptor (CAR) T-cell therapy has demonstrated remarkable efficacy against multiple myeloma (MM). However, several barriers continue to limit the overall effectiveness of this approach, such as high production costs, prolonged manufacturing timelines, safety issues, and the potential for tumor antigen escape due to selective therapeutic pressure. To overcome these challenges, innovative CAR T strategies, such as engineering modular CAR systems, are being explored. These systems utilize adaptor molecules to enable multi-antigen targeting, thereby enhancing specificity, safety, and overall efficiency of CAR T-cell therapy. Notably, CAR T-cells directed against BCMA and SLAMF7 antigens have generated strong and robust antitumor responses in MM therapy.

**Methods:**

To address the limitations of conventional CAR T therapy, we developed a novel modular CAR platform targeted against BCMA and SLAMF7. This was achieved using a split intein-mediated protein splicing mechanism, which allows specific covalent peptide bonds to form between CAR modules. This strategy maintains an almost seamless CAR structure, preserving its overall integrity and functionality. The design of the intein-spliced CAR system (termed "CARtein") was further optimized through advanced protein structure prediction software.

**Results:**

Cells expressing the spliced CARtein constructs, engineered to target BCMA, SLAMF7, or both antigens simultaneously, demonstrated robust and highly specific activation in response to their respective antigens.

**Discussion:**

These results suggest that the CARtein platform is a promising, versatile, and highly specific approach for the modular design and engineering of CARs, enabling multi-antigen targeting while maintaining structural and functional integrity. This modular strategy addresses key limitations of conventional CAR T-cell therapy and may improve both the safety and effectiveness of future MM treatments.

## Introduction

Chimeric antigen receptor (CAR) T-cell therapy has demonstrated remarkably effective long-lasting clinical responses in patients with hematological malignancies, including B-cell leukemia, lymphoma and multiple myeloma (1–4). However, this therapy still needs to overcome many limitations, such as high production costs and long manufacturing periods, or hurdles related to potential lethal toxicity such as cytokine release syndrome (CRS) and neurotoxicity due to CAR overactivation. Other factors that may hinder the efficacy of CAR therapy against hematological cancers are the tumor inhibitory microenvironment and other tumor scape mechanisms. Nevertheless, approaches are being developed in order to improve the antitumor response and safety of CAR therapy, as well as to facilitate the manufacturing process (2, 5).

Despite improvements in survival of multiple myeloma (MM) patients in the last decade due to the development of novel therapeutic alternatives such as monoclonal antibodies or immunomodulatory drugs, it remains largely incurable as most patients eventually relapse (5, 6). In this landscape, CAR therapy has shown promising results in the treatment of patients with relapsed or refractory multiple myeloma. B-cell maturation antigen (BCMA), also known as TNFRSF17, has been the preferred antigen targeted by CAR T-cells directed against MM, as it is highly expressed in most MM malignancies and CAR T-Cell therapies directed against this surface antigen have elicited powerful antitumor responses in relapsed or refractory patients (5, 7–9).

However, many patients exhibit resistance mechanisms after short-term efficacy of CAR T-cell therapy directed against BCMA. One of the most concerning evasion strategies by cancer cells is antigen downregulation under therapeutic pressure, resulting in relapses and poor prognosis during CAR therapy. Therefore, targeting more than one surface protein is an effective approach in order to prevent and/or manage antigen escape. Among the candidates for antigen targeting, SLAMF7 (also known as CS1, CD319 or CRACC) is one of the most promising alternatives. SLAMF7 is a surface antigen upregulated in MM that plays a significant role in the uncontrolled proliferation of malignant cells (5, 10–13). Gogishvili et al., showed that CAR T-cells targeting SLAMF7 by an antigen binding domain based on Elotuzumab exhibited a strong antitumor response (14).

New strategies for targeting multiple antigens in CAR therapy are being developed, such as the tandem design, with a single construct containing two distinct antigen-binding domains or the dual CAR T-cell approach, which is based on the co-expression of two individual CARs targeting different surface proteins in a single T cell (15, 16). In this scenario, engineering of modular CARs containing a cytoplasmic signaling and an extracellular moiety that will capture tailored recognition domains with affinity for different surface antigens is an attractive alternative for multiple targeting, as it will decrease construct size, thus improving transduction efficiency and improve safety as recognition domains can be easily discontinued and/or blocked (17, 18). Multiple modular CAR strategies are currently being explored with promising results, including systems like the UniCAR platform or the anti-FITC CAR strategy. However, most of these approaches rely in transient interaction between labeled antibody and antigen in CAR-modified cells. Nonetheless, new systems are emerging, such as the SpyCatcher-SpyTag platform and its derivatives based in the formation of an isopeptide linkage (19–23).

Inteins are proteins capable of performing through successive nucleophilic displacement reactions, an autocatalytic process, known as protein splicing, in which, they can join adjacent residues via a covalent peptide bond that can be considered almost scarless, since inteins are excluded from the final peptide and only exteins (3aa flanking sequences) remain in the mature construct. Split inteins function as orthogonal pairs composed of an N-terminal fragment (IntN fused to N-extein) and a C-terminal fragment (IntC fused to C-extein), which self-catalyze sequence-specific trans-splicing. This process precisely ligates the flanking 3aa exteins through a covalent peptide bond, while excising the intein, enabling scarless fusion of proteins attached to either or both intein fragments (24–27). Intein-mediated protein splicing has already proven to be a promising platform for protein modification in gene and cell therapy. For instance, Han et al., 2017 successfully generated bispecific IgG antibodies through a technology platform based on split inteins. Moreover, a similar system was explored by Ray et al., 2023 in which they efficiently modified target membrane proteins for live cell application (28–31).

**Figure 1:**
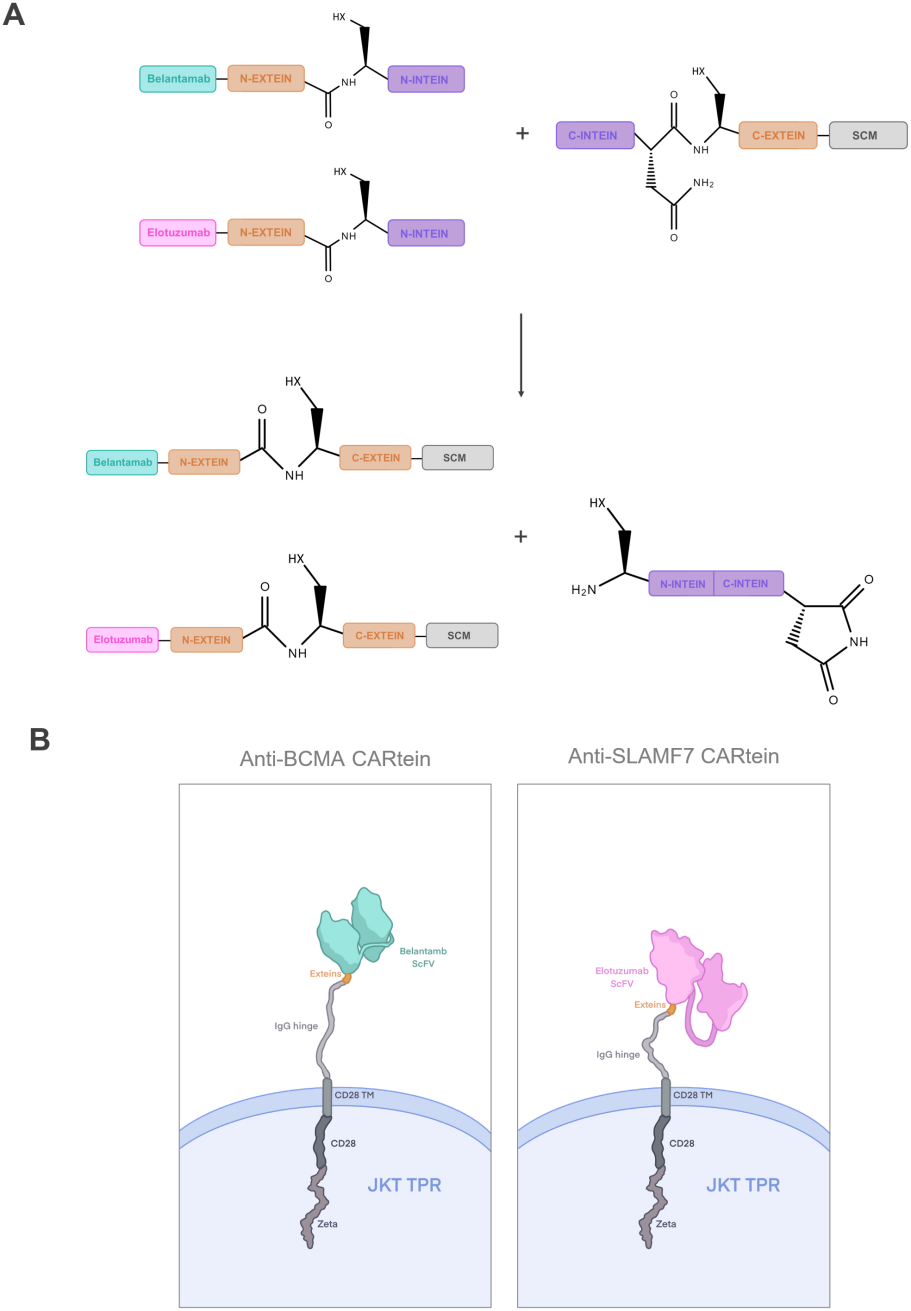
Dual targeting CARtein strategy. **(A)** Schematic representation of CARtein modules before and after split intein-mediated protein splicing. **(B)** Representation of Jurkat (JKT) triple parameter reporter (TPR) expressing the final anti-BCMA and anti-SLAMF7 CARtein constructs. JKT TPR, Jurkat-TPR cells.

In this study, we designed a dual CAR approach for targeting BCMA and SLAMF7 in MM cells through the development of a modular CAR platform based on split intein-mediated protein splicing, which we named CARtein system. For this purpose, we separated the chimeric antigen receptor in two distinct modules, each containing orthogonal split inteins as shown in **Figure 1A**. The signaling CARtein module (SCM) was fused to the C-terminal intein IMPDH-1 (27) while the antigen recognition modules, containing scFv targeting either SLAMF7 or BCMA, were fused to the orthogonal N-terminal part. The spliced anti-BCMA and SLAMF7 CARtein constructs are shown in **Figure 1B**. As previously described, split inteins are not present in the final CARtein construct, except for the residual exteins, consisting in a short sequence of 3 residues each. Herein, we demonstrate that the CARtein system targeting BCMA, SLAMF7 or both antigens simultaneously, allows Jurkat T cells to elicit a specific activation response against MM cells, which makes the CARtein platform an attractive approach for multiple targeting through modular CAR T-cell therapy, as the junction between modules is mediated by a covalent peptide linkage and the proteins responsible for binding are not present in the final CAR sequence. Moreover, we show that structure prediction software, such as ColabFold based on the deep learning model AlphaFold2, can prove a useful tool for rational CAR design (32, 33).

## Materials and methods

### Protein structure prediction and protein docking

In order to facilitate and simplify the rational design of the CARtein modules, we performed protein structure predictions of these constructs. All protein folding predictions were run with ColabFold (ColabFold (RRID: SCR_025453) open-source software (32). In order to improve the accuracy, the prediction of sequences containing parts of the IMPDH-1 split intein were carried out with the crystal structure of gp41–1 intein (PDB id: 6QAZ) used as template. First, we predicted the tridimensional structure of the extracellular domain of the signaling CARtein module (SCM) containing a CD28 transmembrane (TM) domain and an IgG_1_ spacer followed by the C-terminal part of IMPDH-1 split intein (including the C-intein and C-extein). Then, we predicted the same structure without the C-terminal part of IMPDH-1 with the aim of assessing how much the split intein impacts on the final structure. The same strategy was employed to predict the antigen recognition modules folding. These modules included either an anti-BCMA (Belantamab) or an anti-SLAMF7 (Elotuzumab) scFv fused to the N-terminal region of IMPDH-1. This analysis was also extended to constructs lacking the split intein.

For the purpose of evaluating whether the partners of IMPDH-1 split inteins in the CARtein modules would have no steric hindrance for performing protein splicing, the antigen recognition modules directed against BCMA or SLAMF7 were docked with the SCM using HADDOCK2.4 (HADDOCK (RRID: SCR_019091) modeling platform (34). Moreover, we performed a prediction of the antigen-CARtein complexes after protein splicing with ColabFold, using the structure of CD28 transmembrane domain as template (PDB id: 7VU5), for comparing the interaction between the scFv and the extracellular domain (ECD) of BCMA (H3BMB5_HUMAN: 1-69aa) or SLAMF7 (SLAF7_HUMAN:23-247aa) without the residual exteins (GGG-SIC).

Additionally, we predicted the structure of two well-established modular CAR approaches using the same scFv and SCM components: (i) the SpyTag/SpyCatcher system, in which the Belantamab or Elotuzumab scFvs were fused to SpyTag and docked to a SpyCatcher domain (22) inserted upstream on the SCM, keeping a similar design to previously described constructs (21); and (ii) the SUPRA CAR-based platform, utilizing SYNZIP1 and SYNZIP2 heterodimeric coiled-coil domains as modular interaction interfaces (35). For the SUPRA constructs, SYNZIP1 was fused to the scFv via a flexible 35-aa glycine/serine linker keeping the same architecture previously described in the SUPRA CAR system (36), and SYNZIP2 was fused to the SCM. SpyTag/SpyCatcher complex predictions were performed with ColabFold, guided using the crystal structure of SpyTag/SpyCatcher covalent complex (PDB id: 4MLI) as a docking template. The anti-SLAMF7 SUPRA CAR-based complex prediction was carried out with ColabFold as well, while the anti-BCMA SUPRA CAR-based construct in complex with the antigen was conducted by AlphaFold server (37). All protein structures and complexes were analyzed and rendered with the 3D protein imager interface (38).

### Cell culture

Human Embryonic Kidney (HEK Lenti-XTM 293T) (Clontech) cell line was used for lentiviral vector production. A Jurkat T JE6.1 (Jurkat, RRID: CVCL_0367) cell subline known as Jurkat-TPR (Triple Parameter Reporter) expressing eGFP, CFP and mCherry, respectively governed by NFAT, NFκB and AP-1 promoters was established by Prof. Steinberger’s group (39) and previously used by us and others to study CAR T-cell activation signaling (40). The human multiple myeloma (MM) cell line MM.1S (RRID: CVCL_8792) was used as SLAMF7+/BCMA+ target in activation signaling assays. Lastly, a chronic myelogenous leukemia K562 cell line (RRID: CVCL_K562) expressing BCMA and/or SLAMF7 was generated to be used as targets in activation signaling assays. All human cell lines were obtained from American Type Culture Collection (ATCC, Manassas, VA).

Human Embryonic Kidney (HEK Lenti-XTM 293T) (Clontech) as well as Jurkat-TPR cell lines were cultured in DMEM GlutaMax medium (Gibco) supplemented with 10% fetal bovine serum (FBS) (Gibco), 1% sodium pyruvate, 2 mM L-glutamine, 10 mM HEPES, 50 µM 2-mercaptoethanol (Invitrogen, Carlsbad, CA, USA), 100 units/mL penicillin–streptomycin (Invitrogen,Carlsbad, CA, USA) and 50µg/ml Gentamicin (Gibco) at 37 °C and 10% CO_2_. MM.1s cell line was cultured in RPMI 1640 medium (Gibco) equally supplemented at 37 °C and 5% CO_2_. Genetically modified K562 cells were cultured in Iscove’s Modified Dulbecco’s Medium (IMDM) (Gibco) supplemented the same way as DMEM GlutaMax and RPMI media at 37 °C and 5% CO_2_.

### Design of CARtein constructs

The signaling CARtein module (SCM) contained the same spacer, transmembrane and intracellular domains as our previously reported ACE2-CAR, including a CD3ζ intracellular domain, a CD28 transmembrane domain mutated for improved membrane expression, a CD28 co-stimulatory domain, and a human IgG_1_ heavy chain spacer spaning hinge, CH_2_ and CH_3_ domain, mutated to prevent FC receptor activation (40). Upstream SCM, the C-terminal IMPDH-1 split intein (including the C-intein and C-extein) was engineered (27), preceded by a Twin-Strep-tag^®^ (tST) connected through a flexible (GGGGS)_2_ linker and a hIgKVIII leader sequence. The SCM construct was synthetized and codon-optimized by GeneArt™ (Thermo Fisher Scientific Inc., Carlsbad, CA, USA) and cloned via a LR Gateway™ reaction into the lentiviral vector pHRSINcPPT CEW, downstream a SFFV promoter.

Two different antigen recognition modules were generated to interact and bind to the SCM via a peptide bond mediated by split intein protein splicing. Antigen recognition modules contained the complementary N-terminal IMPDH-1 split intein and either an anti-SLAMF7 scFv (Elotuzumab) or anti-BCMA scFv (Belantamab). Both antigen recognition modules were followed by a flexible (GGGGS)_2_ linker and an in-frame KDEL sequence, responsible for sequestering these modules in the endoplasmic reticulum (ER) until the interaction of the N-terminal IMPDH-1 with its C-terminal partner in the SCM, occurs ensuing in the mature CAR molecule. These antigen recognition modules were synthetized and codon-optimized by GeneArt™ (Thermo Fisher Scientific Inc., Carlsbad, CA, USA) and cloned via a LR Gateway™ reaction into the expression lentiviral vector pLEX_307 (a gift from David Root (Addgene plasmid # 41392; http://n2t.net/addgene:41392; RRID: Addgene_41392), under the control of an EF-1α promoter.

### Design of SLAMF7 and BCMA surface antigens

SLAMF7 and BCMA extracellular and transmembrane domains coding sequences were synthetized by GeneArt™ (Thermo Fisher Scientific Inc., Carlsbad, CA, USA) and then cloned via a LR Gateway™ reaction into the expression lentiviral vector pLEX_307, governed by an EF-1α promoter.

### Lentiviral vector production

HEK Lenti-XTM 293T cells were used to produce all lentiviral supernatants. These cells were co-transfected with the corresponding transfer vector, together with plasmids pMD2.G, encoding for the Vesicular Stomatitis Virus G protein (VSVG) (RRID: Addgene_12259) and pCMVΔR8.91 (RRID: Addgene_202687), encoding for HIV-1 GAG and POL proteins. Polyethylenimine (PEI)-mediated transfection was performed in OptiMEM™ medium (Thermo Fisher Scientific Inc., Carlsbad, CA, USA) according to the method optimized by Tang et al., 2015 (41). Lentiviral supernatants were collected at 48 and 72 h, centrifuged for cell debris removal and concentrated with a lentivirus concentrator solution containing 40% (W/V) PEG-8000 and 1.2M NaCl, according to the 4×Lentivirus Concentrator Solution protocol facilitated by the University of Texas M.D. Anderson Cancer Center (RRID: SCR_004699). The high-titer virus-containing pellets were frozen at -80°C until use and viral titers were determined by evaluating transduction efficiency in Jurkat cells.

### Cell transduction

Jurkat-TPR or K562 cells were first cultured for 24h in fresh media, then virus-containing pellets were thawed and resuspended in culture media. 48h after transduction, cells were centrifuged and plated in fresh media. K562 cells (5x10^5^ cells/ml) transduced with either BCMA or SLAMF7 expressing lentiviral vectors, were selected by addition of 0.5μg/ml puromycin Dihydrochloride (Gibco). SLAMF7+ K562 cells were then transduced and re-selected for BCMA ECD expression. Surface antigens expression in K562 cells was analyzed by FACs 72h after selection.

Jurkat-TPR cells (3x10^5^ cells/ml) transduced with lentiviral vectors containing SCM constructs, were selected 48h after transduction by culturing in 40 μg/ml of Blasticidin S HCl (Gibco). SCM expression in Jurkat-TPR transduced cells was evaluated by FACs 72 h after selection. SCM+ Jurkat TPR cells were then transduced by either or both antigen recognition modules directed against BCMA and/or SLAMF7. After 48h, transduced cells were selected through the addition of 0.125ug/ml of puromycin Dihydrochloride (Gibco) to the cell culture media at a final concentration of 3x10^5^ cells/ml. CARtein module expression in Jurkat-TPR cells were evaluated by FACs 72h after selection.

### T cell activation signaling assays

Jurkat-TPR cells expressing the SCM and the final CARtein sequences were co-cultured (10^5^ cells/well) with MM.1s target cells at different target to effector (T:E) ratios (1:1, 1:5 and 1:10) in 96-well flat-bottomed plates in a final supplemented DMEM volume of 200ul. Cells were then incubated at 37°C and 10% CO_2_. T cell activation signaling was measured at three different time points: 0h (unstimulated), 24h and 48h after stimulation. The signal of Jurkat-TPR promoter reporters as well as CD69 upregulation were analyzed for each time point.

To further assess CARtein specificity, target K562 cells either expressing BCMA or SLAMF7 ECD, or both (SLAMF7+/BCMA+ K562) were co-cultured with Jurkat-TPR cells expressing the SCM and the final CARtein sequences (10^5^ cells/well) at T:E ratio of 1:1 in 96-well flat-bottomed plates at a final volume of 200ul of supplemented DMEM. Cells were incubated at 37°C and 10% CO_2_. Jurkat-TPR activation signaling was equally measured at three different time points: 0h (unstimulated), 24h and 48h after stimulation. Signal of NFAT and NFκB promoter reporters as well as CD69 upregulation were analyzed for each measurement.

### Flow cytometry

Expression of SCM and anti-BCMA or anti-SLAMF7 post-splicing CARtein sequence in Jurkat-TPR cells were evaluated by staining with Strep-Tactin^®^XT DY-649 (Iba-Lifesciences, #2-1568-050), Brilliant Violet 421™ anti-human IgG Fc Antibody (Biolegend, San Diego, CA, USA) and Biotinylated Recombinant Protein L Protein, His,Avitag™ (ACROBiosystems, RPL-P81Q7) followed by PE-conjugated streptavidin™ (Thermo Fisher Scientific Inc., Carlsbad, CA, USA). Zombie Violet™ Fixable Viability Kit (Biolegend, San Diego, CA, USA) was used to assess live cells, except for cells stained with Brilliant Violet 421™ anti-human IgG Fc Antibody, for which Zombie NIR™ Fixable Viability Kit (Biolegend, San Diego, CA, USA) was used.

In order to prevent unwanted binding of antibodies to human FC receptors in K562 and MM.1s cells, they were previously blocked with FcR Blocking Reagent, human (Cat# 130-059-901, RRID: AB_2892112, Miltenyi Biotec, Cologne, Germany). Expression of BCMA and SLAMF7 surface antigens in MM.1s cells and transduced K562 cells were evaluated by staining with APC anti-human CD269 (BCMA) Antibody (Cat# 357506, RRID: AB_2562889, Biolegend, San Diego, CA, USA) and PE/Cyanine7 anti-human CD319 (CRACC) Antibody (Cat# 331816, RRID: AB_2565237, Biolegend, San Diego, CA, USA) respectively. For T cell activation signaling assays analysis, Jurkat-TPR cells were stained with anti-human CD69-APC (Cat# 310909, RRID: AB_314844) and CD3-PerCP/Cy5.5 antibodies (Cat# 100218, RRID: AB_1595492) both from Biolegend (San Diego, CA, USA), in order to discriminate CARtein-TPR cells from target cells and evaluate CD69 expression. Cells were also stained with Zombie Violet™ Fixable Viability Kit (Biolegend, San Diego, CA, USA) for live cells determination. Flow cytometry was performed on Cytek^®^ Aurora 5L 16UV-16V-14B-10YG-8R spectral cytometer (RRID: SCR_019826). Data was collected using SpectroFlo software (Cytek SpectroFlo RRID: SCR_025494, Cytek Biosciences) and analyzed using FlowJo software V10.8.1 (RRID: SCR_008520, TreeStar Inc., Olten, Switzerland). Flow cytometry gating strategy for both T cell activation signaling assays is shown in **Supplementary Figure S1**.

### Statistical analysis

Statistical analyses were performed with GraphPad Prism v.9.0.2 (RRID: SCR_002798, GraphPad, La Jolla, CA, USA). Data are presented as the mean ± standard errors of the mean (SEM) from independent triplicates performed on different days to account for batch effects. Two-way ANOVA was used for statistical comparison among multiple groups followed by a Tukey’s multiple comparison test. *p* value of less than 0.05 was considered significant. *p* value significance levels are indicated in the figures (*p < 0.05, **p < 0.01, ***p < 0.001, ****p < 0.0001).

## Results

### CARtein structure prediction

In order to evaluate beforehand whether the integration of split intein modules into mature chimeric antigen receptor would distort protein folding, therefore impairing its functionality, we performed structure and complex predictions with ColabFold software, based on the deep learning model of AlphaFold2 (32, 33). AlphaFold2-based software is capable of performing highly accurate protein structure predictions, and is emerging as a powerful and useful tool in rational protein design, including chimeric antigen receptors development (42, 43).

**Figure 2:**
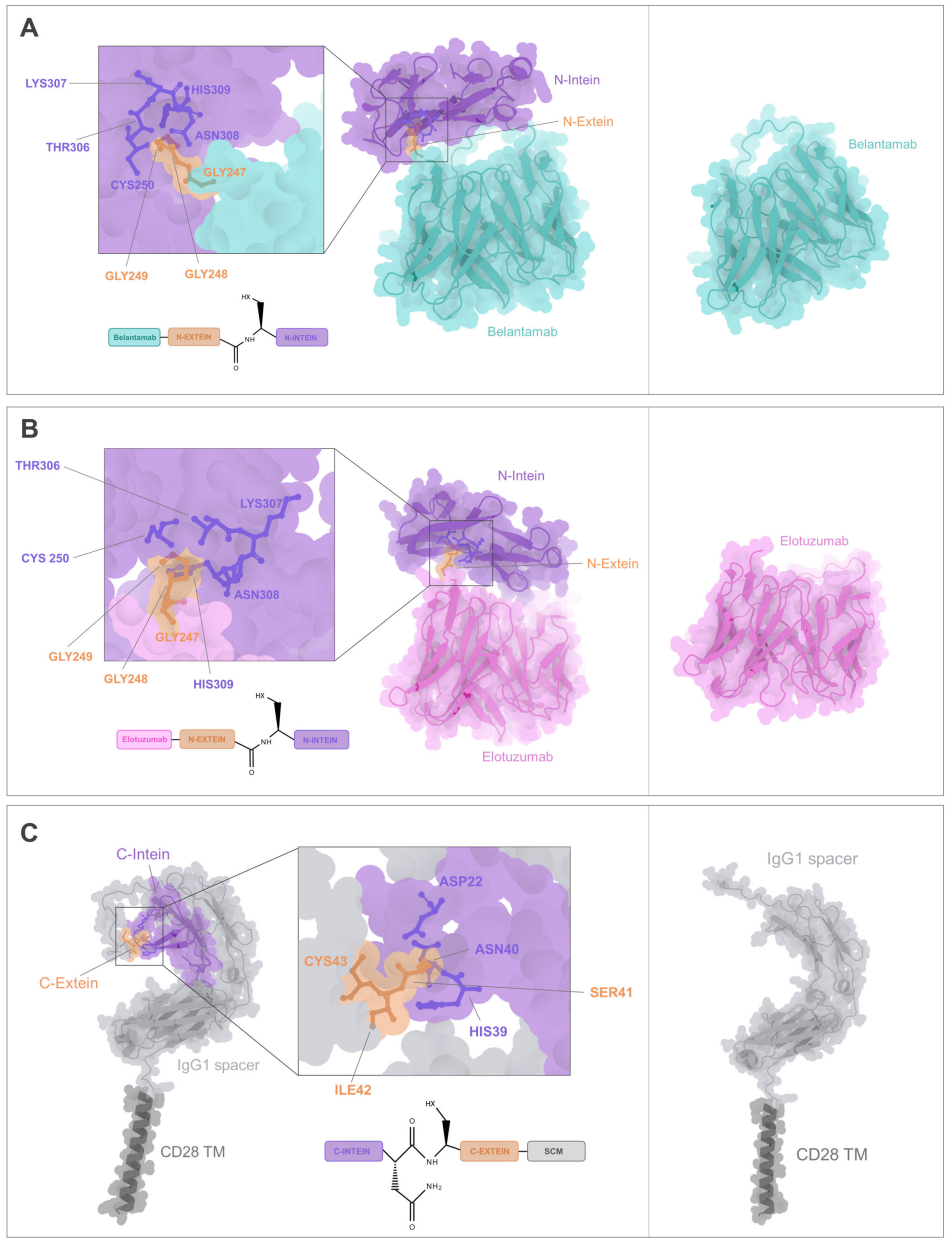
CARtein modules structure prediction. **(A)** Folding prediction of anti-BCMA antigen recognition module containing the scFv (Belantamab) fused to the N-terminal part of IMPDH-1 split intein (including the N-intein and N-extein domain) compared to the unmodified anti-BCMA scFv. **(B)** Structure prediction of anti-SLAMF7 antigen recognition module consisting in the scFv (Elotuzumab) fused to the N-terminal part of IMPDH-1 split intein compared to unmodified anti-SLAMF7 scFv. **(C)** Structure prediction of the extracellular domain of the Signaling CARtein module (SCM) spaning C-terminal intein IMPDH-1 (including the C-intein and C-extein domain) followed by IgG_1_ spacer domains and CD28 TM compared to the SCM without intein. Relevant residues for split intein-mediated protein splicing are shown.

First, we wanted to evaluate whether the integration of IMPDH-1 split intein parts into our CARtein modules would affect their folding and therefore, functionality. We found that structure predictions of anti-BCMA (Belantamab) and anti-SLAMF7 (Elotuzumab) scFvs contained within the antigen recognition modules were sufficiently well-preserved after the addition of the N-terminal part of IMPDH-1(**Figures 2A, B**) when compared with the unmodified scFv predicted conformation. Furthermore, the N-terminal section of the split intein was accessible to the split intein partner, and the structural configuration of complementarity-determining region (CDR) loops of both Belantamab and Elotuzumab were well-preserved. Moreover, we performed a folding prediction of the extracellular and transmembrane domains of the signaling CARtein module (SCM), which comprises a CD28 transmembrane (TM) domain and the IgG_1_ spacer coupled to the C-terminal part of IMPDH-1 split intein (**Figure 2C**). The SCM folding conformation in the presence of the intein moiety was well conserved when compared to the sequence in the absence of intein. In addition, IMPDH-1 C-terminal part is also accessible to its split intein partner.

**Figure 3:**
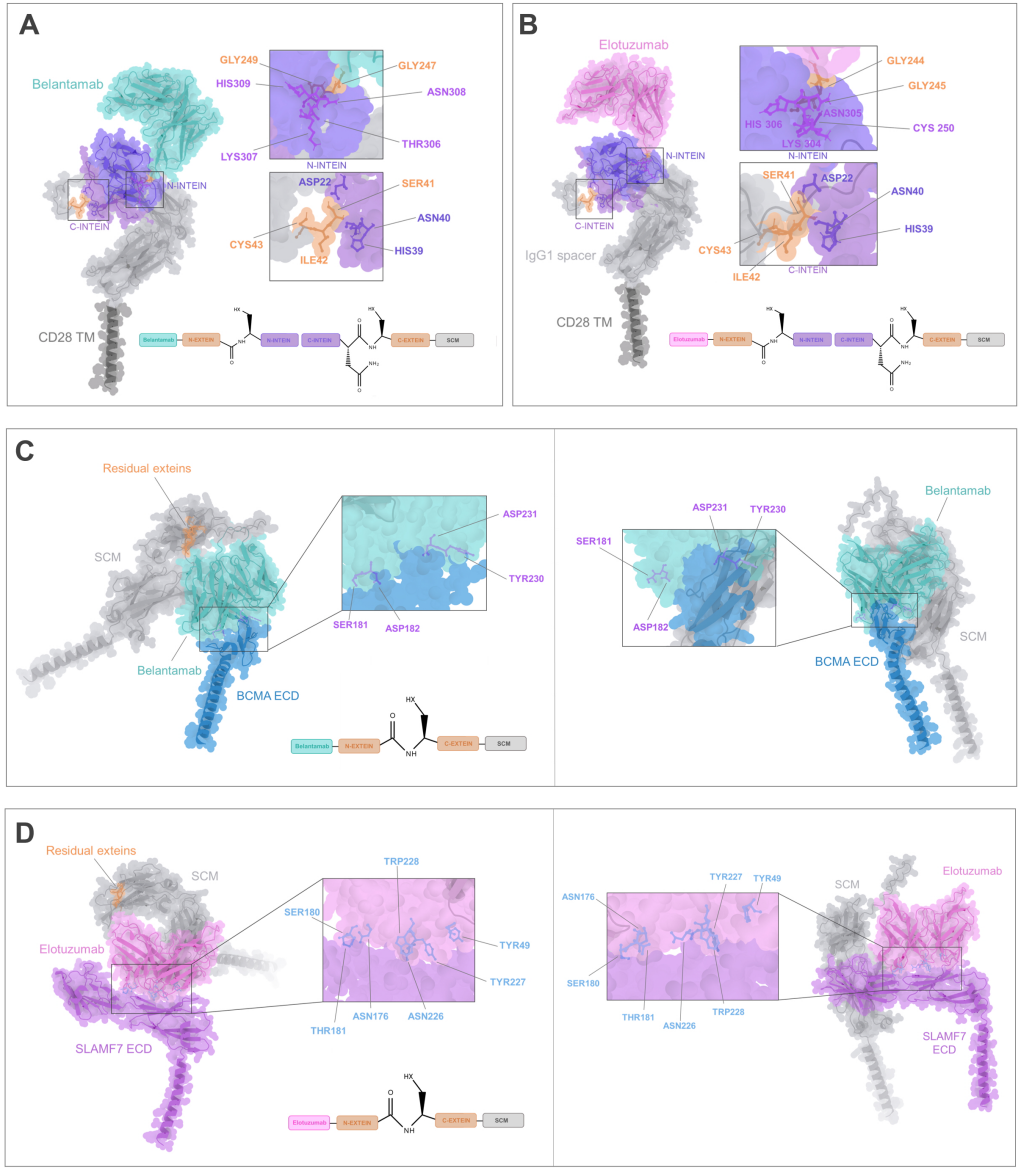
CARtein complexes prediction. **(A)** Docking of the predicted structures for anti-BCMA antigen recognition and SCM modules, with key residues for intein-mediated protein splicing being shown. **(B)** Docking of the predicted tridimensional structures of anti-SLAMF7 antigen recognition and SCM modules, with highlighted relevant residues for split intein-mediated protein splicing. **(C)** Complex prediction of post spliced anti-BCMA CARtein bound to BCMA ECD, compared to a CAR without exteins. scFv residues proximal to the antigen are highlighted. **(D)** Complex prediction of post-spliced anti-SLAMF7 CARtein bound to SLAMF7 ECD compared to a CAR devoid of residual exteins. Elotuzumab residues proximal to the antigen are shown.

The following step was to analyze whether the IMPDH-1 split intein partner embedded within the CARtein modules were sufficiently accessible for protein splicing. For this purpose, the predicted structure of the antigen recognition modules directed against BCMA or SLAMF7 was docked with the predicted conformation of the SCM using HADDOCK2.4 protein-protein docking platform (34). It was noted that in both cases, the anti-BCMA and anti-SLAMF7 antigen recognition modules to exhibited an accessible split intein N-terminal region, thereby facilitating engagement with its C-terminal partner contained within the SCM, with no discernible major steric hindrances (**Figures 3A, B**).

Following the split intein-mediated protein splicing process, the N-extein (GGG) domain of IMPDH-1, which is embedded within the antigen recognition module, will be covalently linked to the SCM C-extein (SIC) through a peptide bond, resulting in the exclusion of the N-intein and C-intein complex from the final anti-BCMA or SLAMF7 CARtein sequence. Accordingly, to assess the potential influence of residual exteins, encompassing six residues (GGG-SIC), on the spliced anti-BCMA and anti-SLAMF7 CARtein structure on scFv-mediated surface antigen recognition, we conducted a prediction of the antigen-CARtein complexes following protein splicing with ColabFold. Structure prediction was performed on the extracellular domain (ECD) of BCMA and SLAMF7, in complex with post-protein spliced CARtein, directed against either BCMA or SLAMF7. Additionally, CARtein constructs lacking residual exteins were analyzed. (**Figures 3C, D**). Comparison of the complex structure prediction between CARtein constructs, in the presence or absence of intervening exteins, suggests that antigen proximal residues in Belantamab and Elotuzumab maintain their relative proximity. Consequently, concluded that scFv functionality, and therefore CAR activity, should not be adversely affected.

**Figure 4:**
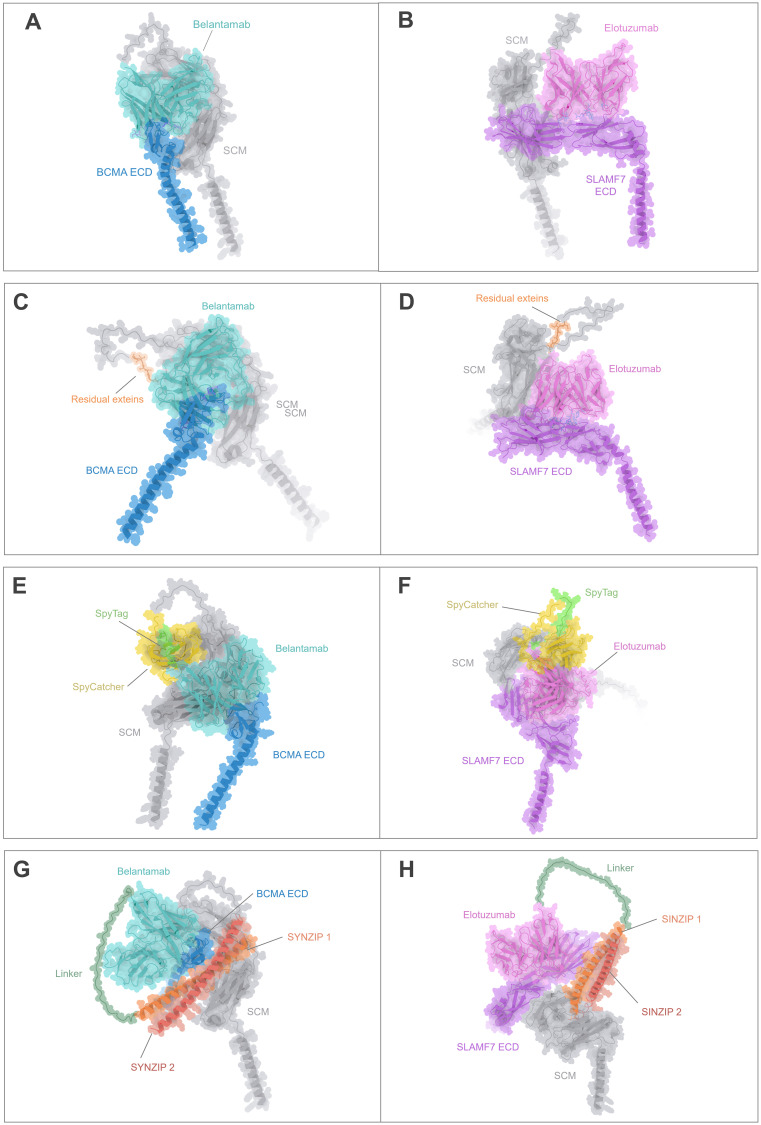
Structural comparison of the CARtein design and other modular CAR architectures in complex with the antigen. **(A, B)** Predicted structures of unmodified anti-BCMA and anti-SLAMF7 CARs in complex with their respective extracellular domains (ECDs). **(C, D)** Predicted post-splicing anti-BCMA and SLAMF7 CARtein complexes comprising residual exteins (GGGSIC) fused to the antigen-binding scFvs. **(E, F)** Predicted SpyTag/SpyCatcher-based modular CAR complexes for anti-BCMA and anti-SLAMF7. **(G, H)** Predicted anti-BCMA and anti-SLAMF7 SUPRA CAR system-based complexes depending on SYNZIP1-SYNZIP2 coiled-coil dimerization incorporating a 35-aa glycine/serine linker between the scFv and SYNZIP1. All models preserve the same SCM, scFvs, and antigen sequences.

To further evaluate the structural integrity of mature CARtein constructs and compare them with other modular CAR designs, we performed additional complex predictions. We compared complexes of anti-BCMA and anti-SLAMF7 unmodified CARs with the extracellular domain (ECD) of their respective antigens (**Figures 4A, B**) and the post-spliced CARtein complexes including the residual exteins (GGG-SIC) resulting from intein-mediated ligation (**Figures 4C, D**). As mentioned before, structural comparisons indicate that the presence of the six-residue extein linker does not noticeably affect the interaction between the scFvs and their respective antigens.

We then performed structure predictions of alternative modular CAR designs employing either SpyTag/SpyCatcher or coiled-coil (SUPRA-based architecture) interactions to mediate antigen recognition module attachment. SpyTag/SpyCatcher-based CARs (**Figures 4E, F**) exhibited an overall bulkier configuration due to the interdomain covalent interaction, while the SUPRA CAR-based complexes (**Figures 4G, H**), which incorporated SYNZIP1/2 and a flexible linker, displayed elongated and more extended conformations. Importantly, in all modular CAR systems analyzed, the scFv maintained an orientation compatible with antigen binding. However, the compactness and minimal distortion observed in the CARtein constructs relative to the native CARs suggest that post-splicing CARteins may offer improved structural fidelity.

Collectively, these results support that CARtein constructs retain a highly native-like conformation after intein-mediated assembly, outperforming other modular systems in terms of structural preservation and minimal interdomain interference. Furthermore, the short extein linker, encompassing a GGGS sequence, may contribute favorable flexibility that supports productive antigen binding without compromising CAR stability or recognition geometry.

### CARtein system design and establishment of CARtein-TPR cells and K562 antigen expressing cells

**Figure 5:**
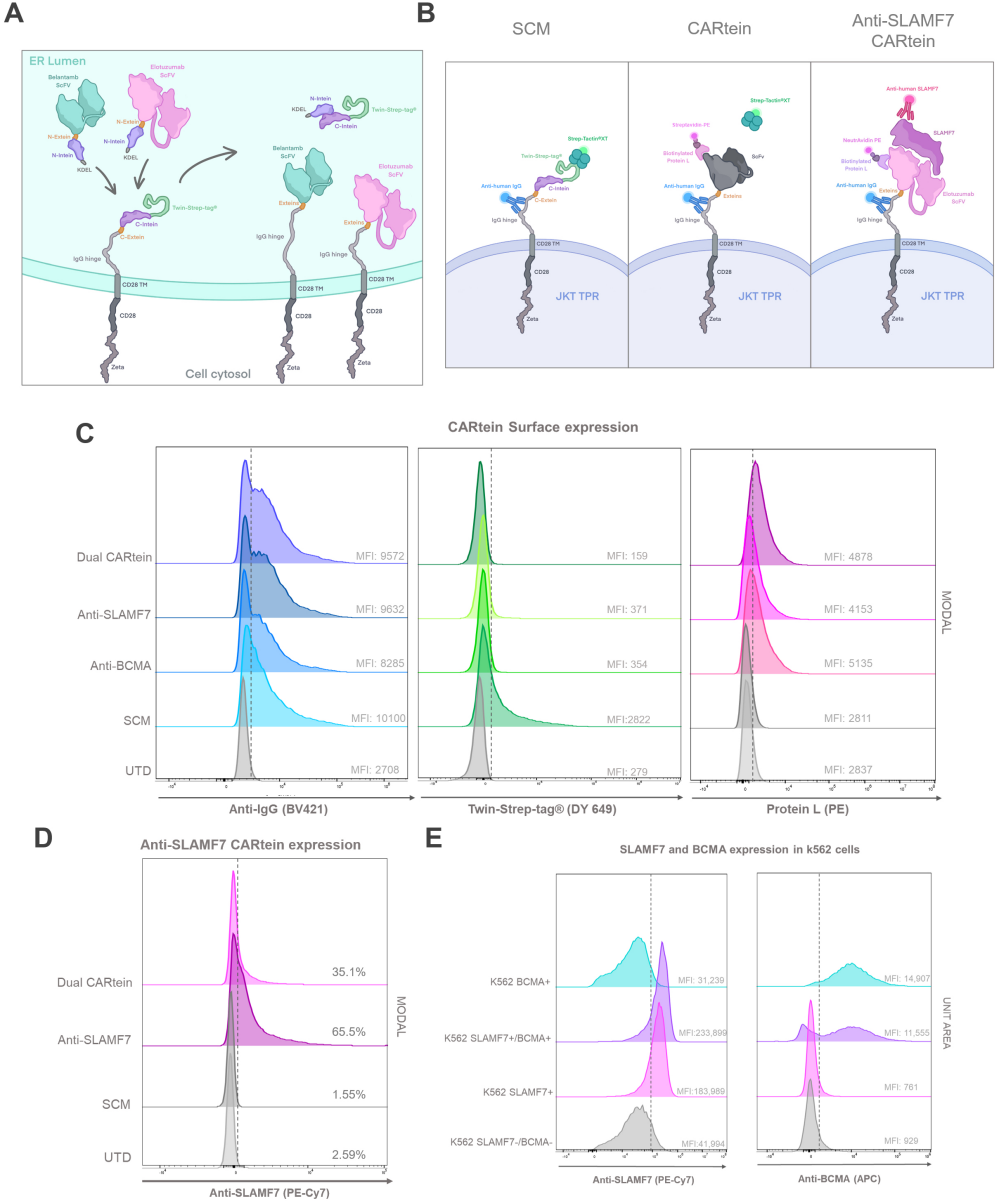
CARtein maturation and expression after split intein-mediated protein splicing. **(A)** Representative illustration of anti-BCMA and anti-SLAMF7 CARtein modules before and after intein-mediated CARtein splicing in the ER. **(B)** Illustrative representation of the staining strategy performed for the evaluation of SCM (Streptactin) mature whole CARtein (Protein L) and SLAMF7 CARtein expression. **(C)** Flow cytometry histogram overlays of SCM and spliced CARtein expressing unpermeabilized Jurkat-TPR cells. **(D)** Flow cytometry histogram overlay of Elotuzumab staining in SCM and anti-SLAMF7 or Dual CARtein-TPR cells. **(E)** Histogram overlay of BCMA and SLAMF7 expression in transduced k562 cells with either or both surface antigens. UTD, untransduced cells. JKT TPR, Jurkat-TPR cells.

In order to generate split intein-mediated modular chimeric antigen receptors (CARteins) against BCMA and SLAMF7, three different CARtein modules were designed, including two distinct antigen recognition modules targeting each surface antigen, both containing the IMPDH-1 N-terminal sequence (encompassing the N-intein-extein), and one signaling CARtein module (SCM), which contained the C-terminal intein (including the C-intein-extein), followed by the IgG_1_ spacer, CD28 transmembrane, and intracellular domains and a CD3ζ intracellular domain (40). A Twin-Strep-tag^®^ (tST) sequence was positioned right after the hIgKVIII leader sequence, upstream the C-terminal intein, for easy detection of the un-spliced SCM module (**Figure 5A**).

Antigen recognition modules comprised the scFv (either Belantamab targeting BCMA or Elotuzumab targeting SLAMF7) followed by the N-terminal IMPDH-1 and an in-frame Lys-Asp-Glu-Leu (KDEL) sequence to retain these modules within the endoplasmic reticulum (ER) until protein splicing via split inteins occurred, as KDEL receptors (KDELRs) retro-transport KDEL-bearing proteins from the Golgi to the ER (44). They were designed in such a way that the anti-BCMA and SLAMF7 CARtein constructs following intein-mediated protein splicing, would lose the tST, the KDEL sequence and the split inteins, except for the six extein residues (GGG-SIC) conforming the final CARtein sequence. As a consequence of KDEL removal, the spliced CARteins would be able to migrate outside the ER and be expressed on the surface membrane (**Figure 5A**).

All three constructs were cloned into lentiviral vectors and expressed in JKT-TPR cells to easily measure Nuclear factor-κB (NFκB) and Nuclear factor of activated T-cells (NFAT) promoter activation, as the synthetic promoters respectively control expression of reporter CFP and eGFP (39). Jurkat TPR cell line has been previously validated as an efficient platform for the evaluation of CAR functionality (45–48). Jurkat-TPR cells were transduced with lentiviral vectors containing the SCM construct, and then selected through the Blasticidin-S resistance gene. SCM expression was evaluated by staining with an anti-IgG antibody recognizing the IgG_1_ spacer and Strep-Tactin^®^XT- Twin-Strep-tag^®^ (tST). SCM+ Jurkat-TPR cells were transduced with lentiviral vectors coding for antigen recognition modules directed against BCMA or SLAMF7, or both vectors simultaneously (Dual CARtein), and further selected by means of a Puromycin resistance gene. Since IMPDH-1 splicing reaction reaches its optimal temperature at 37°C, the split intein-mediated protein splicing occurred spontaneously under standard Jurkat cell incubation (27).

In order to analyze SCM, anti-BCMA, anti-SLAMF7 and Dual CARtein expression in Jurkat-TPR cells by flow cytometry, a triple staining strategy was carried out. (**Figure 5B**). Cells transduced with SCM alone or together with one or both recognition modules were stained with an anti-IgG antibody recognizing the IgG_1_ spacer, Strep-Tactin^®^XT or Protein L, which recognizes either Belantamab or Elotuzumab VL, followed by PE-conjugated streptavidin™ (49). As evidenced by IgG-staining, the IgG_1_ spacer was highly and consistently expressed among both, cells expressing unspliced SCM and those expressing any post spliced mature CARtein. As expected, Mean Fluorescence Intensity (MFI) values corresponding to tST staining in SCM+ Jurkat-TPR cells decreased in anti-BCMA, anti-SLAMF7 or Dual CARtein cells, indicating that intein-mediated protein splicing has occurred in these cells. Moreover, this is supported by the concomitant increase in protein L MFI in post-spliced CARtein cells, in contrast with single transduced cells that only expressed SCM and maintain high tST and low Protein L staining. Notably, anti-SLAMF7, anti-BCMA and Dual CARtein cells exhibited similar MFI values and flow cytometry histogram profiles for anti-IgG, protein L and Strep-Tactin^®^XT staining (**Figures 5B,C**). To evaluate ARM occupancy in dual CAR-expressing cells, we incubated them with soluble SLAMF7 antigen followed by a fluorescent anti-SLAMF7 antibody (**Figure 5B** right panel) targeting an epitope distinct from that recognized by the Elotuzumab-derived scFv in our anti-SLAMF7 ARM (an equivalent non-competing antibody was unavailable for BCMA/Belantamab). This approach allowed detection of unoccupied SLAMF7-binding sites. Dual CARtein cells exhibited significantly reduced anti-SLAMF7 staining intensity compared to cells expressing only anti-SLAMF7 CAR, while total CAR expression (protein-L staining) remained equivalent across samples. The reduced SLAMF7-specific signal confirms that only a subset of SCMs is occupied by the anti-SLAMF7 ARM, while the remainder are bound to the anti-BCMA ARM, demonstrating successful co-assembly of both ARMs and a balanced occupancy with no competitive exclusion.

To assess the specificity of the activation response elicited by CARtein-TPR cells targeting BCMA, SLAMF7 or both surface antigens, K562 cells were transduced with lentiviral vectors expressing BCMA or SLAMF7, and then puromycin selected. To obtain K562 expressing both antigens, after antigen expression and FACS evaluation, SLAMF7+ K562 cells were transduced and re-selected for BCMA expression. As shown in **Figure 5E**, transduced K562 cells with BCMA (BCMA+ K562), SLAMF7 (SLAMF7+ K562) or both surface proteins (SLAMF7+/BCMA+ K562) consistently expressed the corresponding surface antigen.

### Kinetics of CARtein-TPR cells upon SLAMF7 and BCMA stimulation mediated by transduced K562 cells

In order to validate the functionality of cells expressing the different CARtein constructs after split intein-mediated protein splicing, we performed a T cell activation signaling assay, using transduced K562 cells as target. We would measure specific CARtein-driven signaling upon BCMA or SLAMF7 stimulation by analyzing expression of CD69 activation marker as well as NFAT and NFκB promoter activity in JKT-TPR cells (CARtein-TPR cells) (39, 50).

Transduced K562 cells were co-cultured with anti-BCMA, anti-SLAMF7 and Dual CARtein-TPR cells for T cell signaling activation analysis at a target to effector ratio of 1:1. CD69 expression, as well as NFAT and NFκB activity of CARtein expressing cells were evaluated at different time points after stimulation (0h, 24h and 48h). Response of SCM+ cells was evaluated as a control. Untransduced Jurkat-TPR cells (UTD) were used for baseline GFP, CFP or CD69 MFI values. Untransduced K562 cells (SLAMF7-/BCMA- K562) were also co-cultured to evaluate K562 mediated non-specific activation. Additionally mock (unstimulated) CARtein cells were also cultured under the same conditions.

**Figure 6:**
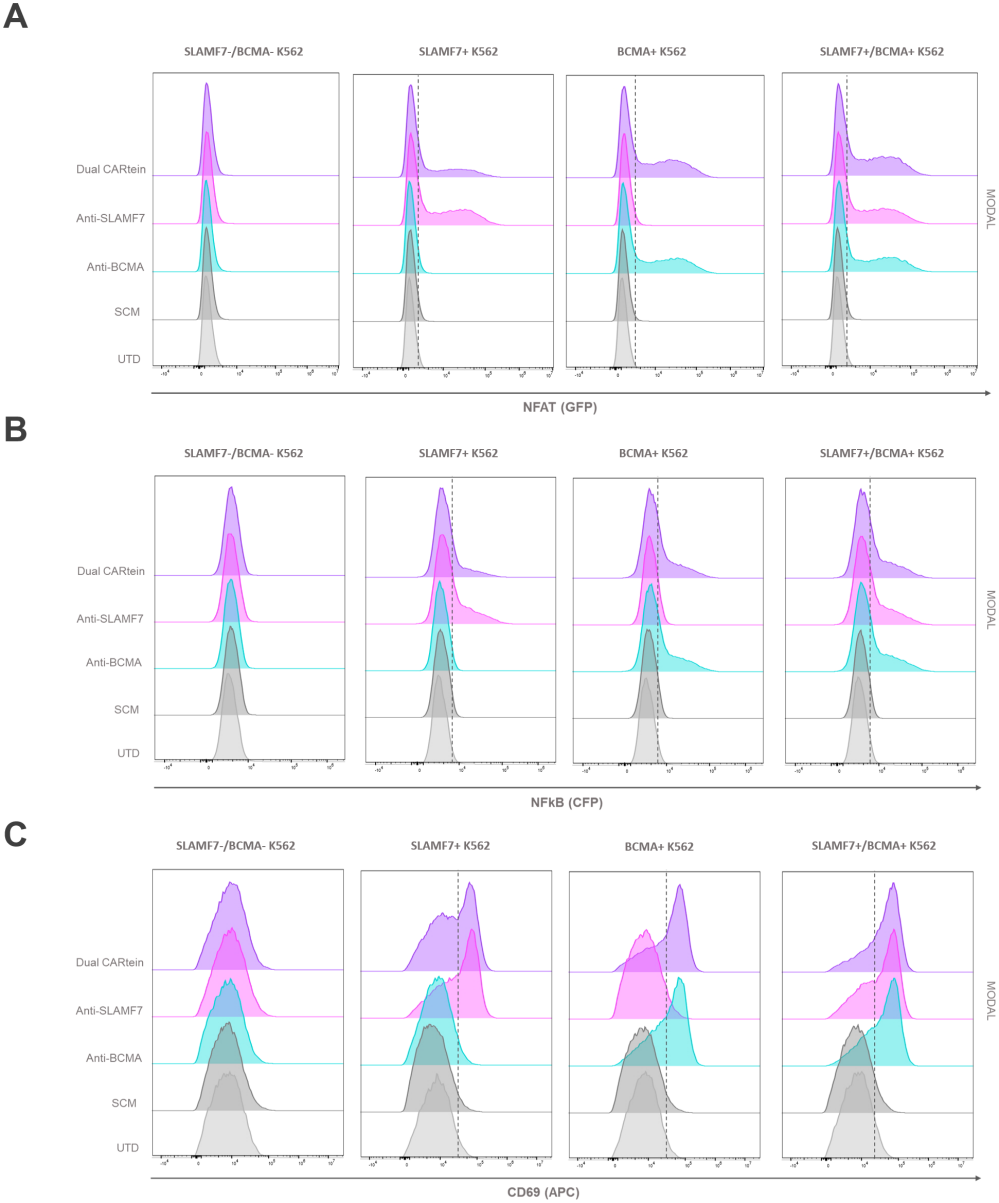
Activation signaling assay of anti-BCMA, anti-SLAMF7 and Dual CARtein-TPR cells co-cultured with k562 cells expressing BCMA, SLAMF7 or both surface proteins. Representative flow cytometry analysis of NFAT **(A)** and NFκB **(B)** promoter reporters, or CD69 **(C)** in CARtein-TPR cells 24h after co-culture with either untransduced (UTD) or CARtein transduced k562 cells in a T:E ratio of 1:1.

Consistent with previous results, anti-BCMA, anti-SLAMF7 and Dual CARtein-TPR cells, elicited NFAT and NFκB activation response directed against their respective targets in transduced K562 cells after 24h (**Figures 6A, B**). Moreover, CARtein cells exhibited relatively high expression of the activation marker CD69 (**Figure 6C**). On the other hand, SCM+ cells did not show CD69 upregulation or SCM-mediated NFAT or NFκB transcriptional activity when coculture with any target cell. Additionally, we did not observe any unspecific activation nor tonic signaling in CARtein-TPR cells or SCM+ cells when co-cultured with untransduced K562 cells. Nonetheless, Dual-CARtein cells exhibited relatively higher GFP and CFP MFI values when co-cultured with SLAMF7+/BCMA+ K562 cells instead of SLAMF7+ K562 cells.

**Figure 7:**
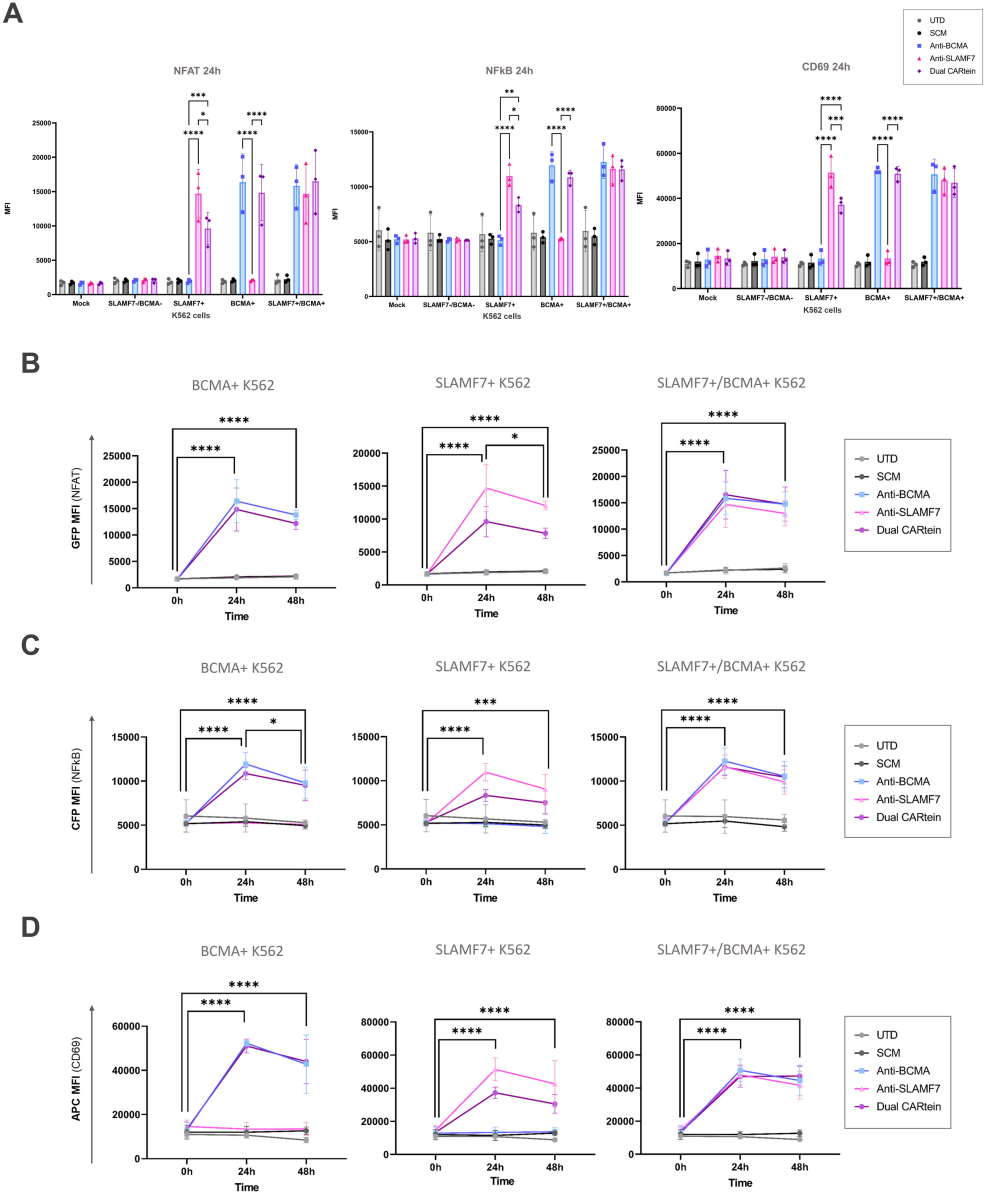
Kinetics of anti-BCMA, anti-SLAMF7 and Dual CARtein-TPR cell activation upon co-culture with k562 cells expressing BCMA, SLAMF7 or both. **(A)** Flow cytometry comparison of NFAT and NFκB reporters and CD69 MFI for CARtein-TPR cells co-cultured with k562 cells expressing BCMA, SLAMF7 or both surface proteins. Each dot represents an independent experimental triplicate. Statistical comparison between anti-BCMA, anti-SLAMF7 and Dual CARtein-TPR cells is indicated (*p < 0.05, **p < 0.01, ***p < 0.001, ****p < 0.0001). NFAT **(B)**, NFκB **(C)** and CD69 **(D)** activation kinetics for anti-BCMA, anti-SLAMF7 and Dual CARtein-TPR cells upon co-culture with either, untransduced (UTD) or CARtein transduced k562 cells. Statistical analysis of Dual CARtein cells is shown for each time condition (*p < 0.05, **p < 0.01, ***p < 0.001, ****p < 0.0001). Data are average ± SEM of three different experimental replicates.

For the purpose of comparing activation of either single or dual CARtein cells when co-cultured with cells expressing BCMA, SLAMF7 or both tumor antigens, we performed a Two-way ANOVA analysis. We did not find statically significant differences when comparing Jurkat-TPR cells stimulated with K562 cells expressing a single antigen or both antigens simultaneously. As illustrated in **Figure 7A**, anti-BCMA and anti-SLAMF7 CARtein cells exhibited high CD69 as well as NFAT and NFκB reporters MFI values (****p < 0.0001) when co-cultured with K562 cells expressing one or both surface proteins. Dual CARtein cells co-cultured with BCMA+ K562 cells elicited a slightly reduced NFAT and NFκB activity, but similar CD69 expression, while upon co-culture with SLAMF7+ K562 cells a significant decrease of CD69 (***p < 0.001), NFAT and NFκB reporters (*p < 0.05) MFI values were observed. Interestingly, in the presence of SLAMF7+/BCMA+ K562 cells, the stimulation of Dual CARtein-expressing cells resulted in slightly higher NFAT activity than single CARtein cells, although not statistically significant. Dual CARtein cells displayed similar CD69 and NFκB reporter MFI values when compared with CARtein cells targeting only one antigen upon SLAMF7+/BCMA+ stimulation. Furthermore, no tonic signaling or SCM+ mediated activation response was observed.

Regarding CARtein activation kinetics, a two-way ANOVA analysis was conducted in order to compare CARtein mediated cell activation at varying time points following stimulation (**Figures 7B–D**). CARtein-TPR cells targeting only one or both surface antigens exhibited a pronounced and significant (**** *p* < 0.0001) GFP and CFP (NFAT and NFκB reporters) upregulation 24h upon stimulation with K562 expressing BCMA, SLAMF7 or both. After 48h, these high GFP and CFP MFI values decreased, although they remained relatively elevated. A similar trend was observed for the CD69 activation marker at 24h and 48h. However, while anti-BCMA and anti-SLAMF7 CARtein cells elicited similar expression patterns, Dual CARtein-TPR cells maintained elevated CD69 expression for 48h.

### Kinetics of T cell activation in CARtein-TPR cells co-cultured with BCMA and SLAMF7 expressing MM.1s cells

**Figure 8:**
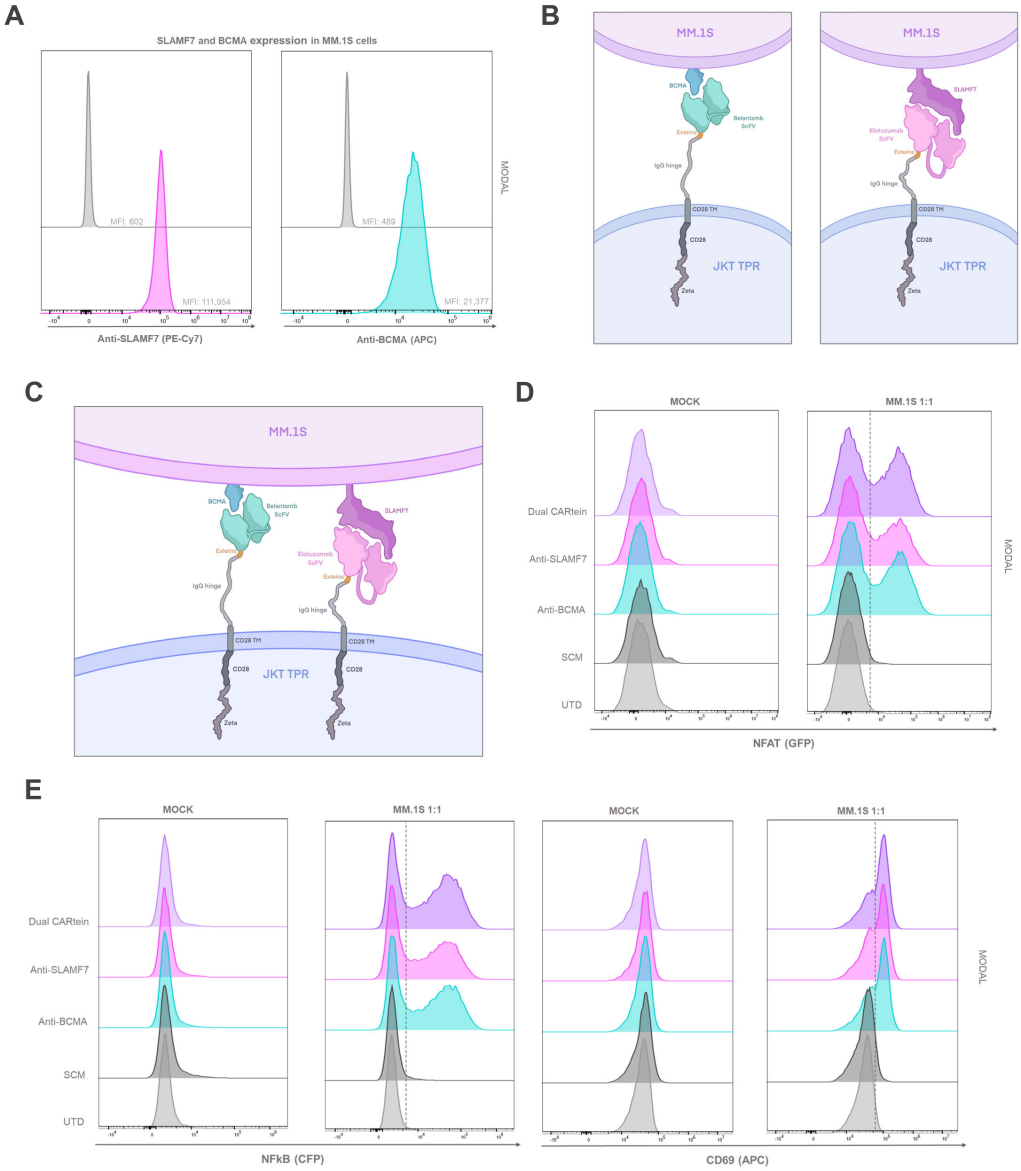
T cell activation signaling assay in CARtein Jurkat-TPR cells co-cultured with MM.1s cells. **(A)** SLAMF7 and BCMA expression in MM.1s cells. Schematic illustration of anti-BCMA or anti-SLAMF7 CARtein **(B)** or Dual CARtein **(C)** cells co-cultured with multiple myeloma target cells. **(D)** Representative flow cytometry analysis of NFAT and **(E)** NFκB activity and CD69 upregulation 24h after co-culture in a T:E ratio of 1:1. UTD, untransduced cells. JKT TPR, Jurkat-TPR cells.

In order to analyze CARtein-TPR cells signaling activation upon interaction with a multiple myeloma cell line, we co-cultured them with MM.1S cells. Prior to MM.1s coculture, we conducted a flow cytometry analysis to evaluate the expression of BCMA and SLAMF7 surface antigens using anti-BCMA and anti-SLAMF7 antibodies for staining. As shown in **Figure 8A**.1s cells exhibited elevated expression of both tumor antigens.

We then co-cultured MM.1s cells, with Jurkat-TPR cells expressing anti-BCMA, anti-SLAMF7 or Dual post-protein splicing CARteins (CARtein-TPR cells) as illustrated in **Figures 8B, C**. Additionally, Jurkat-TPR cells expressing the SCM (SCM+ cells) were also stimulated, in order to assess any potential SCM-mediated tonic signaling. As a control for baseline eGFP and CFP fluorescence signals, MM.1s cells were also co-cultured with untransduced (UTD) Jurkat-TPR cells [Mock (unstimulated)] We then analyzed by flow cytometry CD69, NFAT and NFκB activity at different time points (0h, 24h and 48h) and for different T:E ratios (1:1, 1:5 and 1:10).

As evidenced in **Figures 8D, E**, anti-CD69 expression as well as NFAT and NFκB activity increased 24h after MM.1s stimulation in anti-BCMA, anti-SLAMF7 and Dual CARtein-TPR, exhibiting similar activation MFI profiles at 1:1. Moreover, minimal or negligible response was observed in SCM+ cells, as well as in mock samples, which suggests that CAR-mediated activation is strongly specific with little to none tonic activation in CARtein-TPR or SCM+ cells. In addition, the biparametric flow cytometry analysis (**Supplementary Figure S2**) revealed that most cells exhibiting CD69 upregulation, aligned with those showing higher NFAT or NFκB reporters signals, as well as cells displaying an enhanced NFAT activity with those with higher NFκB MFI values, indicating that most activated cells exhibited a complete signal transduction process.

**Figure 9:**
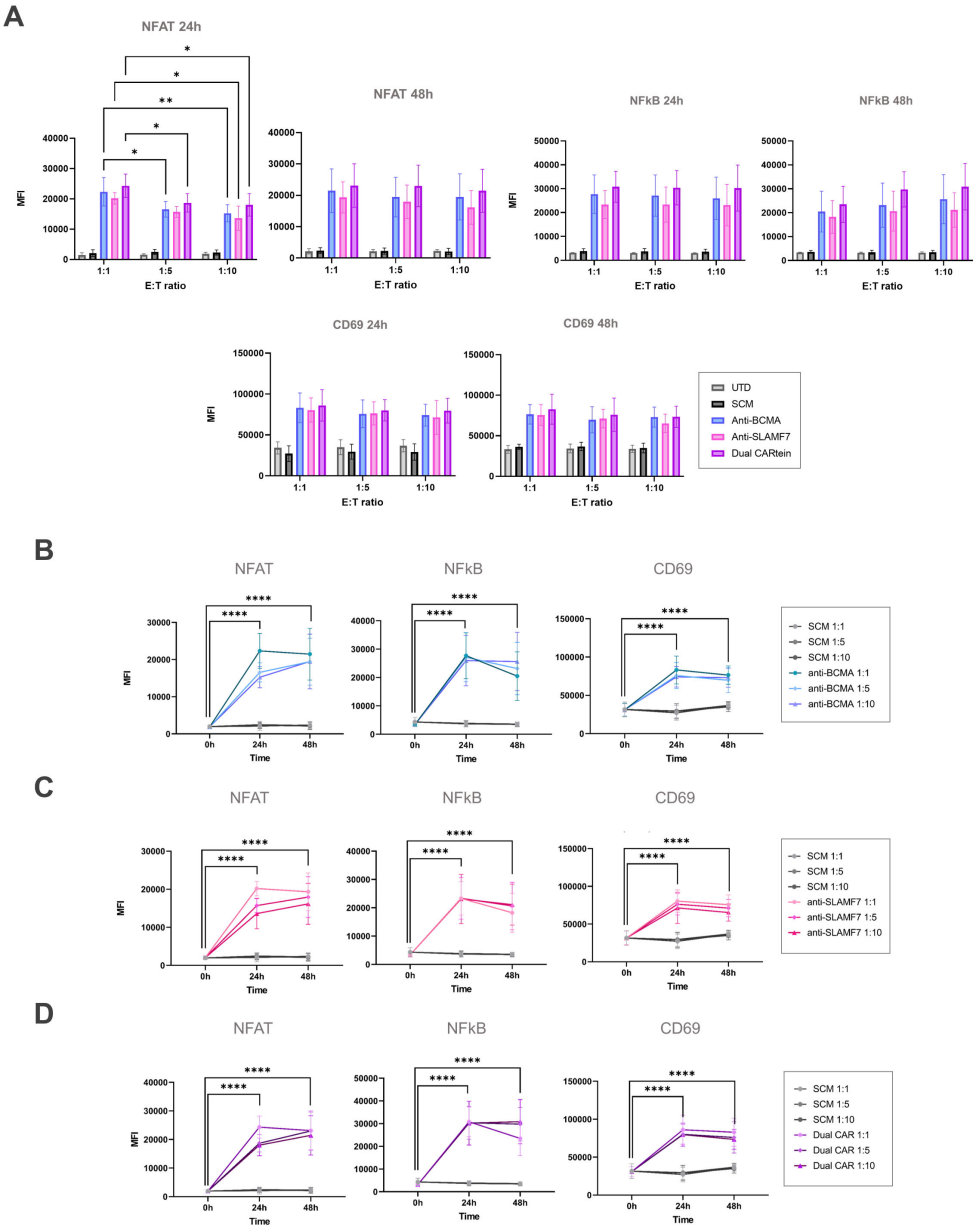
Kinetics of anti-BCMA, anti-SLAMF7 and Dual CARtein-TPR cell activation upon MM.1s stimulation. **(A)** Flow cytometry-based statistical comparison of the mean fluorescence intensity (MFI) of NFAT and NFκB activity reporters and CD69 upregulation after co-culture with MM.1S cells at three different T:E ratios. Statistical analysis between T:E ratios is shown (*p < 0.05, **p < 0.01, ***p < 0.001, ****p < 0.0001). **(B)** NFAT, NFκB and CD69 activation kinetics for three T:E ratios of anti-BCMA, **(C)** anti-SLAMF7 and **(D)** Dual CARtein cells upon stimulation. Statistical analysis of CARtein cells in 1:1 ratio is indicated for each time condition (*p < 0.05, **p < 0.01, ***p < 0.001, ****p < 0.0001). Data are average ± SEM of three different experimental replicates. UTD, untransduced cells.

With the aim of comparing anti-BCMA, anti-SLAMF7 and Dual CARtein-mediated T cell activation upon antigen stimulation at different target to effector ratios (1:1, 1:5 and 1:10), we performed a two-way ANOVA analysis. As illustrated in **Figure 9A**, all CARtein cells elicited a strong, specific and sustained NFAT and NFκB-mediated response as well as CD69 upregulation at different target to effector ratios. We found statistically significant differences among T:E ratios for NFAT reporter (GFP) MFI values in CARtein-TPR cells 24h after stimulation. Anti-BCMA CARtein-mediated NFAT activity moderately decreased at 1:5 (*p < 0.05) and 1:10 (**p < 0.01) ratios compared to 1:1 ratio. The same effect was observed for Dual CARtein cells (*p < 0.05), as well as for anti-SLAMF7 cells (*p < 0.05) between 1:1 and 1:10 ratios. However, NFAT activity analysis performed 48h after stimulation, revealed that CARtein cells exhibited similar MFI values for different T:E ratios for each construct. Interestingly NFκB activation is delayed at 48h in 1:5 and 1:10 ratios when compared to 1:1 ratio. Notably, SCM+ cells showed none or insignificant activation response, suggesting that no unspecific SCM-driven activation is exhibited by CARtein cells. Interestingly, MFI values for CD69 and NFAT and NFκB reporters exhibited by Dual CARtein cells exceeded those of CARtein cells targeting a single antigen in all T:E ratios and for both, 24h and 48h after co-culture, although the difference is not statically significant. This tendency may be indicative of a synergistic effect in T cell signal transduction when cells expressing these CAR constructs target both antigens.

To gain further insight into the activation kinetics of these CARtein-TPR cells, a two-way ANOVA analysis was conducted, comparing different time points for a cell expressing a particular intein-mediated CAR (**Figures 9B–D**). NFAT, NFκB and CD69 MFI values at target to effector 1:1 ratio strongly peak at 24h upon antigen stimulation for anti-BCMA, anti-SLAMF7 and Dual CARtein cells (****p < 0.0001). However, NFAT signal slightly decreases, although no significantly, for all CARtein-expressing cells at 1:1 ratio after 48h. A similar but more pronounced effect can be appreciated for NFκB activity. Moreover, we can observe a delayed NFAT response for all CARtein-TPR cells at T:E ratios 1:5 and 1:10, since they reach their highest MFI values at 48h (****p < 0.0001). Interestingly, anti-BCMA CARtein cells at 1:10 ratio and Dual CARtein cells at 1:5 and 1:10 ratios maintained high NFκB MFI values up to 48h, but not in the remaining cases, in which the signal declined. Regarding CD69 expression, 24h after co-culture, all CARtein-TPR cells exhibit high CD69 MFI values at different T:E ratios. Nonetheless, cells co-cultured at 1:1 ratio display a slightly higher, although no significant, CD69 expression. CD69 activation marker MFI values remained elevated 48h after stimulation, even though a downward trend is appreciated. NFAT, NFκB and CD69 MFI values remained at background levels for SCM+ cells at any T:E ratio or time point.

**Figure 10:**
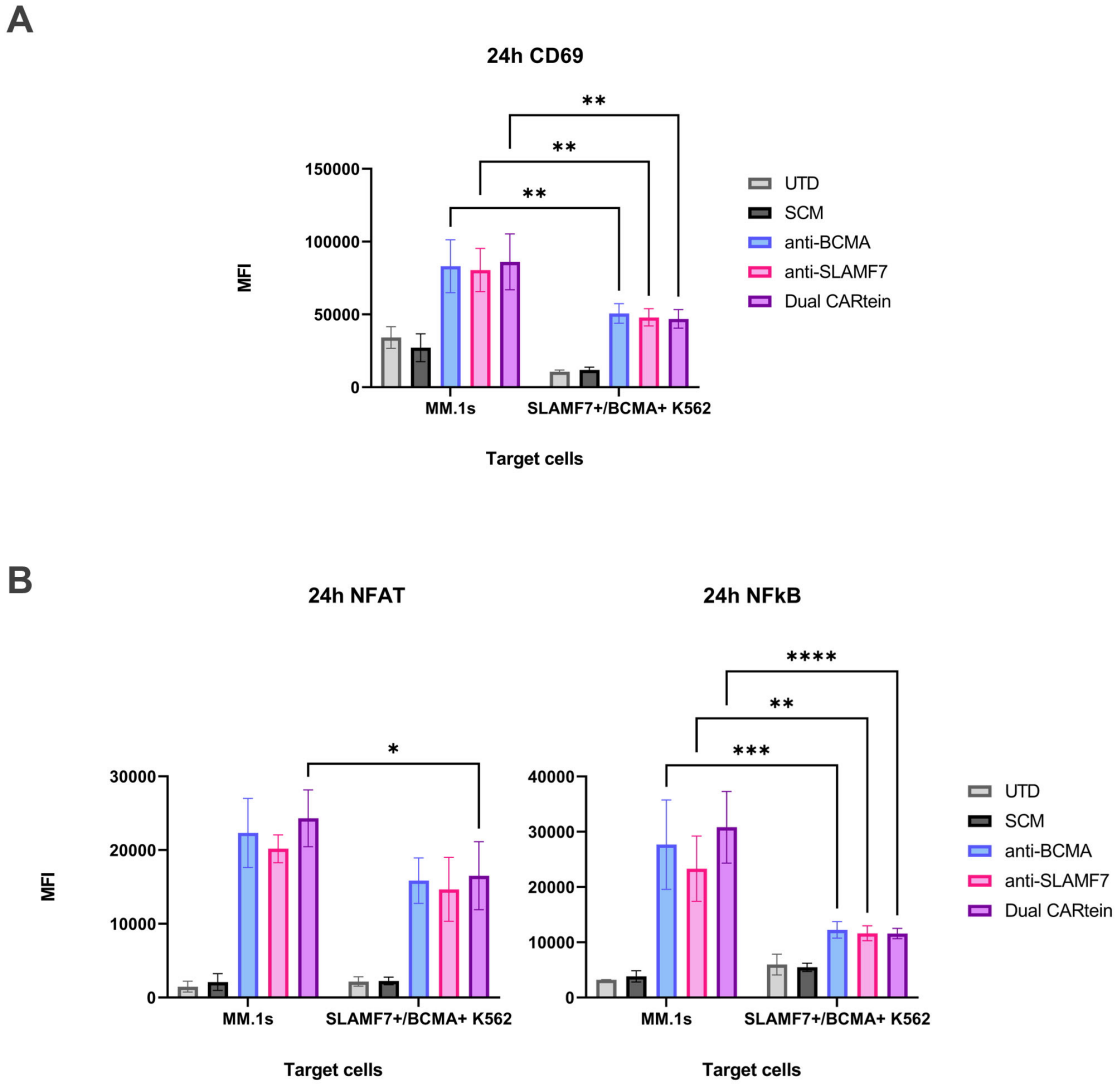
Comparison of CARtein-TPR cell activation upon stimulation with MM.1s or K562 cells expressing BCMA and SLAMF7. **(A)** Flow cytometry-based statistical comparison of the mean fluorescence intensity (MFI) of CD69 activation marker and **(B)** NFAT and NFκB reporter activity 24h after coculture at a target to effector ratio of 1:1. Statistical analysis between MM.1s and SLAMF7+/BCMA+ coculture is shown (*p < 0.05, **p < 0.01, ***p < 0.001, ****p < 0.0001). Data are ± SEM of three different experimental replicates. UTD, untransduced cells.

To obtain a more comprehensive view regarding CARtein-mediated signaling activation in T-cells, we performed a two-way ANOVA analysis, comparing NFAT, NFκB and CD69 MFI values in CARtein-TPR cells when stimulated with MM.1S multiple myeloma cell line, which endogenously expresses BCMA and SLAMF7 proteins, or K562 cells, which have been engineered to express both simultaneously (**Figures 10A, B**). For both cases we conducted the statistical analysis at target to effector ratio of 1:1 and 24h after coculture, as NFAT and NFκB reporters, as well as CD69 marker, show the highest MFI values. Dual CARtein cells exhibit significantly enhanced NFAT (*p < 0.05), NFκB (***p < 0.001) activity and increased CD69 expression (**p < 0.01) when stimulated with MM.1s cells. Moreover, anti-BCMA and anti-SLAMF7 CARtein cells also displayed significantly higher CD69, NFκB MFI values (** p ≤ 0.01) and NFAT after MM.1s coculture.

## Discussion

In this study we have demonstrated successful assembly and antigen-dependent activation of a split intein-mediated CAR approach targeting BCMA and SLAMF7 in MM cells using the Jurkat-TPR cellular model. Moreover, the anti-BCMA scFv Belantamab, which has given promising results for MM treatment as an antibody-drug conjugate (51, 52), has been used for the first time in the context of a CAR construct being highly selective and functional for CAR T cell therapy. For the modular system we have chosen IMPDH-1 intein as it exhibits the highest trans-splicing rates and yields (27, 53), and it has already been shown to function in ER transplicing (53). The dual CARtein strategy proposed herein can be used for the complex integration and expression of two or more independent CAR constructs simultaneously in a single cell, as it has been previously reported that Dual CARs outperform bispecific CARs based on two distinct scFvs linked in tandem or pooled CARs strategies (54), overcoming critical limitations observed in tandem CARs. Tandem CARs tether two scFvs to a single signaling domain forcing co-dependency, where steric hindrance between scFvs may reduce binding efficiency, while for CARtein, each ARM assembles with its own SCM, creating two independent CAR populations per cell, thus preserving full signaling autonomy and allowing for independent spacer optimization, as spacers of different lengths could be included in the ARM instead of SCM when necessary. Although different bispecific CAR approaches targeting BCMA and SLAMF7 have already been developed for multiple myeloma treatment, the Dual CARtein system represents the first modular CAR engineered to target BCMA and SLAMF7 simultaneously, thus offering dual functionality without signal dilution. Leveraging intein-mediated protein splicing, this platform offers unprecedented adaptability, irreversible action, compact design, and minimal toxicity (55–59). The CARtein system overcomes limitations of existing modular CAR platforms by providing irreversible stability with minimal scarring. This is achieved through a permanent peptide bond, chemically identical to native protein bonds, leaving only a 6-amino acid scar (GGGSIC). This scar is comparable in size and impact to cloning artifacts in conventional CARs and can even be further shortened (although such optimization may risks reduced splicing efficiency). Importantly, the residual split intein sequences are no longer part of the CAR construct and exhibit negligible immunological impact, as confirmed by unsuccessful attempts to generate antibodies in animal immunization studies. As for the Twin-Strep tag, it has been used experimentally but is removable in a therapeutic construct. To further enhance safety, we are developing proprietary clearance strategies to eliminate residual intein-derived sequences from circulation. On the other hand, non-covalent systems (SUPRA CAR, UniCAR, etc.) rely on antibody-epitope, receptor-ligand, or leucine zipper interactions that risk dissociation and require continuous adapter infusion, while also leaving bulky scars (e.g., leucine zippers, scFv domains) that may disrupt immune synapse formation and increase immunogenicity. The spycatcher/tag systems: form irreversible covalent bonds but leave a large bacterial-derived scar (138 aa spycatcher + 13 aa spytag) linked by an isopeptide bond, a foreign domain that significantly elevates immunogenicity risk. Biotin-binding cars: introduce non-covalent streptavidin/biotin interactions (bbir/msa2-cars) with risks of interference from endogenous biotinylated proteins, variable valency, and a bulky (~53 kda) and potentially immunogenic avidin/streptavidin scar (60).

The CARtein system also offers versatility in assembly modes, with trans-assembly based on external adapter (ARM) administration for flexible targeting and/or ARM optimization, but also with the possibility of intracellular assembly such as the one shown in our work that is unique to the CARtein system, where transduced ARMs enable self-contained functionality, eliminating adapter infusion entirely, ensuing in a mature CAR with a minimal scar that minimizes anti-CAR immune responses and avoids steric hindrance at the immunological synapse. We have shown that in cells co-expressing both SCM as well as ARM modules, mature spliced CARtein receptors are expressed in the cell surface as staining shown in **Figure 5** are performed in unpermeabilized cells.

The Jurkat-TPR cell line, a cellular model for T cell signaling activation studies, was used to evaluate the functionality and specificity of the split intein-mediated CAR (CARtein) system directed against multiple myeloma. This cellular model, in which the expression of eGFP, CFP and mCherry proteins is respectively governed by NFAT, NFκB and AP-1 transcription factors, has been previously validated for the functional analysis of CAR constructs (45, 47, 61). In this study we show NFAT and NFκB activity, given that AP-1 activation can better be monitored in T cells the human distal ARRE-2 site from the IL-2 promoter used in Jurkat-TPR cells as NFAT reporter as it is dependent on NFAT and AP-1 cooperative binding (62, 63). Additionally, we further evaluated CD69 expression upon antigen stimulation, as CD69 is an early activation marker rapidly expressed on lymphocyte surface upon stimulation (64).

Overall, a strong and specific T cell signaling activation response was elicited upon antigen stimulation of Jurkat-TPR cells expressing the post-spliced anti-BCMA or anti-SLAMF7 CARtein individual constructs, as well as in those expressing both CARtein constructs simultaneously (Dual CARtein-TPR cells). High CD69, NFAT and NFκB reporters MFI values were reported in CARtein-TPR cells upon stimulation with MM.1s and K562 cells transduced with one or both target antigens. Interestingly, when reducing the number of target cells (MM.1s) relative to the number of effector cells (T:E ratios 1:5 and 1:10), CARtein-TPR cells displayed delayed NFAT and NFκB activation compared to the one displayed at 1:1 ratio. However, these cells still exhibited a relatively strong signal transduction despite the significant reduction in the number of target cells, suggesting that our CARtein system is highly sensitive and selective, even at low concentrations of cancer cells, which will allow strong responses against low or moderate tumor burdens. Nonetheless, an exacerbated response of CAR T cells against cancer cells can lead to toxic systemic cytokine levels, and may result in significant adverse effects, such as the cytokine-release syndrome (CRS) or immune effector cell-associated neurotoxicity syndrome (ICANS) (65, 66). However, previous studies have reported that CAR-mediated activation in T cells upon antigen binding can be tuned by optimizing CAR molecular density, which may decrease CAR T cells activation response if necessary (48, 67) a feature that can be optimized by means of the modular CARtein system either by controlling the expression of the recognition module by means of a inducible promoter or by adding in trans proteins spanning the signaling module.

Regarding signaling activation kinetics in CARtein-TPR cells, when co-cultured with MM.1s cells at 1:1 ratio, JKT-TPR cells expressing the anti-BCMA, anti-SLAMF7 or both CARtein molecules exhibited similar kinetics trend for NFAT reporter and CD69 expression as previously reported for ACE2-CAR-Like cells, although NFAT activation follow a faster kinetic with maximal stimulation at 24h instead of 48h. However, NFκB kinetics were closer to one previously described to be exhibited by JKT-TPR cells after anti-CD3+ anti-CD28 stimulation, with a strong peak 24h after antigen engagement followed by a decline in MFI values at 48h. Nonetheless CARtein cells at target to effector 1:5 and 1:10 ratios displayed closer NFAT, NFκB and CD69 kinetics to the one exhibited by ACE2-CAR cells (40). These findings may indicate that, although our CARtein cells exhibit activation kinetics similar to ACE2-CAR-like expressing cells, with delayed and sustained NFAT activity and CD69 expression at 1:1 ratio, CARtein cells may elicit an activation response that more closely resembles that of T-cell receptor (TcR) than CAR-like cells, as it is widely accepted that anti-CD3+ anti-CD28 stimulation mimics TcR engagement and signal transduction (68). Nevertheless, when stimulated with a reduced number of target cells (1:5 and 1:10 ratios), CARtein-TPR cells elicited a delayed and maintained NFAT and NFκB activation kinetics, similar to the activation response reported by Rydzek et al., using ROR1-specific CAR cells bearing two reporter genes that induce the expression of reporter proteins under the government of NFAT and NFκB (45).

Remarkably, when co-cultured with K562 cells expressing either BCMA, SLAMF7, or both antigens, CARtein cells exhibited a decreased NFκB activity compared to those stimulated with MM.1s. Moreover, CD69 MFI values were also significantly reduced, while NFAT MFI values remained relatively similar. In addition, Dual CARtein-TPR cells stimulated with MM.1s cells, exhibited higher NFAT, NFκB and CD69 MFI values than cells expressing only one of the spliced CARtein molecules. This tendency may be indicative of a synergistic effect due to the total antigen density obtained by targeting both antigens on target cells because on the effector cell the total CARtein density is the same regardless of the signaling module. This enhanced effect in T cell signaling activity could also explain the fact that Dual CARtein-TPR cells exhibited higher NFAT and NFκB reporters MFI values when co-cultured with SLAMF7+/BCMA+ K562 cells compared to K562 cells expressing only BCMA or SLAMF7 surface antigens. Fernández Larrea et al., reported that an anti-BCMA and anti-GPRC5D dual CAR approach based on bicistronic vectors encoding two independent CARs outperformed the anti-myeloma efficacy of a mono-targeted pooled CAR approach (54). However, further experimentation is required to elucidate the exact mechanisms underlying this enhanced antitumor response.

MM.1s-mediated co-stimulation could explain the enhanced synergetic effect when CARtein cells are stimulated with the multiple myeloma cell line rather than genetically modified K562 cells expressing target antigens. This theory is further reinforced by the fact that Jurkat T cell line JE6.1 consistently express CD28 receptor and the multiple myeloma cell line, MM.1s expresses CD86 (B7-2) ligand. It is described that CD28 activation is mediated by the binding to its ligands, CD80 and CD86, which eventually leads to the induction of NFκB-dependent expression of the anti-apoptotic protein BCL-XL, which is a key survival mechanism in T-cells (69–71). Moreover, it has been reported that in the absence of co-stimulation, TCR signaling induces a PLCγ-dependent and prolonged increase in cytosolic Ca2+ concentration and the transcriptional induction of NFAT, which is consistent with the reduction of NFκB activity in CARtein cells when co-cultured with transduced K562 cells instead of MM.1s cells, while NFAT-mediated activation remained relatively similar (72).

One of the current limitations in CAR T cell therapy is ligand independent tonic signaling, which can lead to poor antitumor response, impaired survival and T cell exhaustion. One of the mechanisms that could be responsible for tonic signaling is the instability of scFvs in CAR molecules, promoting self-aggregation and therefore signaling via CD3ζ (73–76). IgG_1_-derived spacers have also been reported to mediate CAR tonic signaling, as cells expressing FcγR I and II could interact with IgG_1_ CH_2_ region (73, 77). However, neither CARtein-TPR cells nor SCM+ cells have exhibited evidence of significant tonic signaling, which implies that none of the mentioned tonic signaling mechanisms are triggering an off-target response. These results are consistent with the absence of significant tonic signaling in our previously reported ACE2-CAR-like cells, which shares with the CARtein constructs a mutated IgG_1_ spacer that does not bind Fc receptors (40, 78). As for the off-tumor toxicity risks for CARtein constructs targeting SLAMF7 and BCMA, they are expected to be identical to conventional CAR-T therapies using the same humanized scFvs, as the mature CARtein receptor is structurally equivalent except for a 6-amino acid scar.

It is also noteworthy that in this study we have employed *in silico* analysis based on protein and complex structure prediction software in order to evaluate whether the integration of split inteins into the scFv or CAR signaling module would negatively impact their structure and hence, functionality. For this purpose, we have used ColabFold software, based on the highly accurate deep learning model AlphaFold2 (32, 33). Not only were we able to predict the structure of the CAR signaling and antigen-binding modules comprising split inteins moieties, but also the ability of said inteins to interact with each other and thus catalyze their reaction. Moreover retention of antigen binding capacity, of spliced CARteins bearing residual exteins was also predicted, as the scFv-binding pocket exhibited minimal alteration. These findings, together with previous results, suggest that protein structure prediction is a valuable and powerful tool for the rational design of modular CARs and other chimeric proteins (23, 42, 79).

Furthermore, unlike conventional modular CAR designs that depend on transient scFv-tag conjugate interactions with modified CAR constructs, the innovative CARtein system employs an intein-mediated protein splicing mechanism that establishes a covalent peptide bond between the N- and C-exteins, involving only three specific residues (19, 80). While alternative modular CAR approaches—such as the SpyTag-SpyCatcher system or the SNAP-CAR platform—also utilize covalent linkage strategies through isopeptide bonds, they present significant limitations (20, 81–84). The CARtein system’s distinct advantage lies in its unique modular attachment mechanism that leaves an exceptionally minimal residual “scar” of merely six amino acids after interaction. This remarkable characteristic substantially reduces the probability of interference with antigen binding, a critical concern in CAR functionality. The split intein-mediated CAR platform proposed in this study effectively addresses these limitations while maintaining optimal binding efficiency (27, 85). In addition, Tornabene et al., reported no signs of intein-related toxicity in retinal gene therapy performed on mice, pigs and human retinal organoids, in which they used adeno-associated viral (AAV) vectors encoding fragments of target proteins followed by split intein sequences in order to overcome issues related to limited AAV cargo capacity (28).

The CARtein^®^ system enables creation of “off-the-shelf” SCM-expressing T-cell banks that can be cryopreserved and rapidly armed with disease-specific ARMs for optimization. Additionally, already optimized ARMs can either be infused or permanently transduced. The system allows for manufacturing efficiency, decoupling a general SCM production from specific ARM design. It will also allow for sequential re-targeting as patients could receive multiple courses with different ARMs without additional T-cell harvests, including ARMs against escape antigen administered to existing SCM^+^ cells. Simultaneous dual targeting to address tumor heterogeneity is also possible as well as tapered dosing, gradually reducing ARM frequency to manage chronic toxicity while preserving SCM^+^ cells. The CARtein system is also relevant for CRS mitigation as system-level activity can be dynamically regulated by halting ARM administration, as mature CARs in the surface are cleared within 24–48 hours and replaced by inactive SCM. For rapid inactivation, the SCM can be engineered to include a secondary C-terminal intein module positioned downstream of the primary intein. This module remains intact post-splicing. Administration of a cognate N-intein effector (lacking scFv domains) then triggers excision of the ARM via intein-mediated splicing, enabling rapid CAR disassembly, a safety switch unattainable with conventional CARs. In conclusion, the CARtein system presents a flexible, versatile, and powerful platform for assembling modular CARs, offering an extensive range of potential combinations that can be further expanded and integrated with diverse strategies, enabling optimization of CAR constructs and the treatment of a broader number of malignancies, thus, providing a valuable resource for universal CAR T cell therapy. While this study establishes proof-of-concept for CARtein^®^ assembly, signaling, and modular functionality in engineered cell lines, demonstrating tumor-cell lysis and cytokine release profiles in primary human T cells remains a critical next step for translational validation that we are currently pursuing. Additionally, SCM transgenic animals or SCM expressing human cells in humanized mice will streamline ARM and CAR optimization processes for preclinical studies in tumor models, while in parallel “off-the-shelf” SCM-expressing T-cell banks that could be further manipulated for allogeneic therapy can be used as a universal CAR strategy and approved in clinical trials, with an independent approval for specific ARM that can be either infused or co-transduction for fixed dual-targeting.

## Data Availability

The raw data supporting the conclusions of this article will be made available by the authors, without undue reservation.
