# Supplementary material for: Dual targeting of BCMA and SLAMF7 with the CARtein system: chimeric antigen receptors with intein-mediated splicing elicit specific T cell activation against multiple myeloma
Source: Front Immunol. 2025 Jul 31;16:1613222. doi: 10.3389/fimmu.2025.1613222 (PMC12350263; doi:10.3389/fimmu.2025.1613222)
Supplement: Supplementary file 1 [file DataSheet1.pdf]

Supplementary Material

Supplementary Figure 1

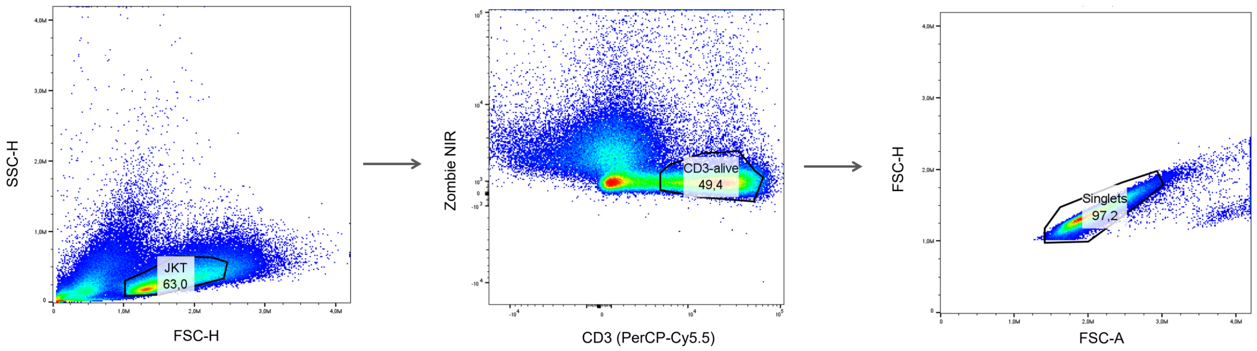

Figure S1. Gating strategy for flow cytometry analysis of CARtein-TPR cells in T cell activation signaling assays. JKT, Jurkat cells.

Supplementary Figure 2

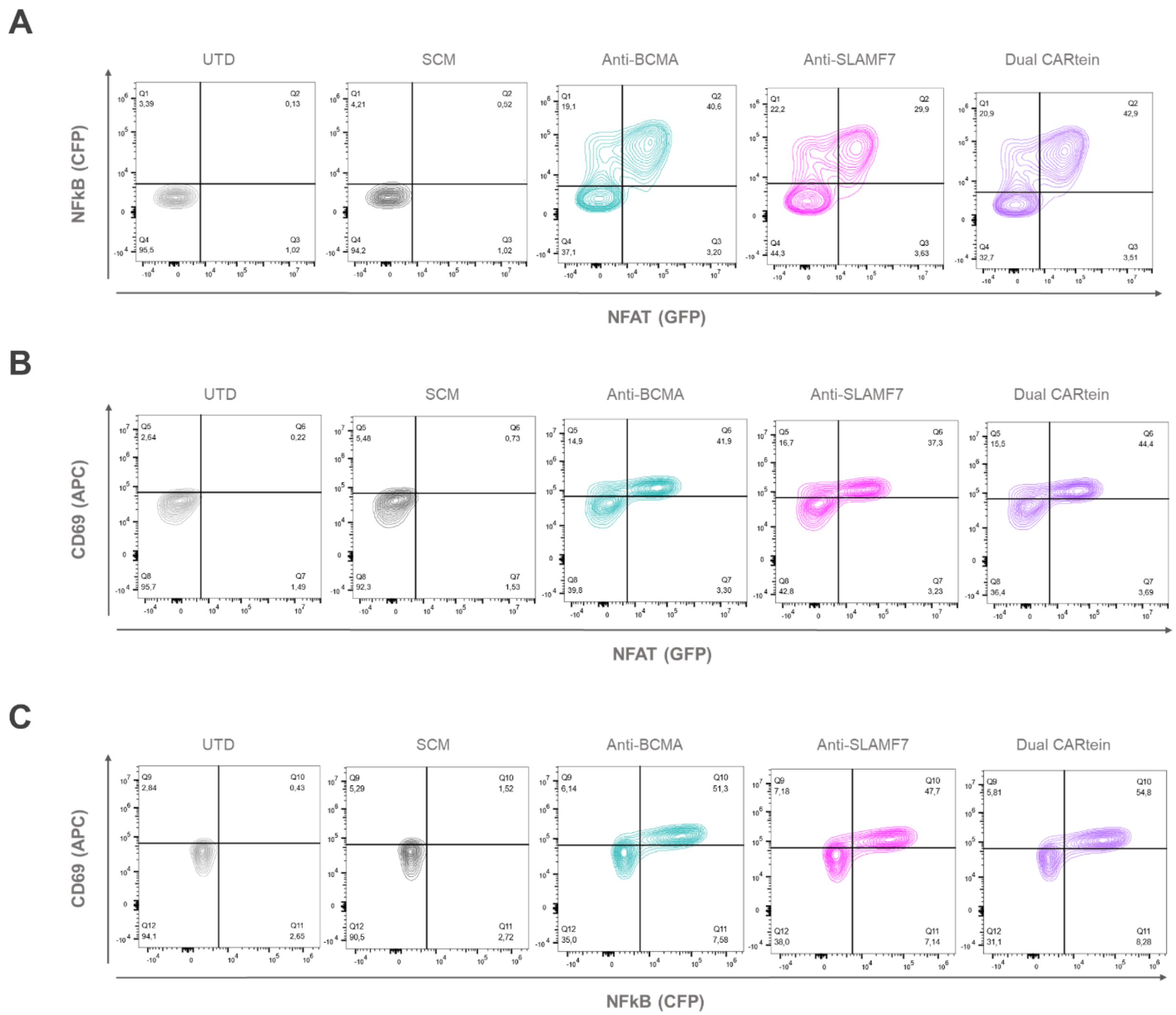

Figure S2. Biparametric flow cytometry analysis of CARtein-TPR cells activation signaling assay after 24h from co-culture with MM.1s cells. **(A)** NFκB and NFAT activity in CARtein-TPR. **(B)** CD69 and NFAT evaluation in CARtein-TPR cells. **(C)** CD69 and NFκB evaluation in CARtein-TPR cells. CARtein cells were co-cultured with MM.1s cells in a T:E ratio of 1:1. UTD, untransduced cells.
